# Lactate metabolism and lactylation in female reproductive diseases: From metabolic rewiring to biomarkers and translational therapeutics

**DOI:** 10.1002/ctm2.70717

**Published:** 2026-06-15

**Authors:** Jiajun Qiao, Yue Xiao, Yi Cheng, Weihai Xu, Jing Shu, Shishi Li

**Affiliations:** ^1^ Reproductive Medicine Center The First Affiliated Hospital, Zhejiang University School of Medicine Hangzhou Zhejiang China; ^2^ School of Basic Medical Sciences and Forensic Medicine Zhejiang Provincial People's Hospital, Affiliated People's Hospital, Hangzhou Medical College Hangzhou Zhejiang China

**Keywords:** biomarker, female reproductive diseases, lactylation, lactate metabolism, translational therapeutics

## Abstract

**Background:**

Lactate is increasingly recognized not only as a metabolic end product of glycolysis but also as a signaling metabolite that links metabolic reprogramming to epigenetic and transcriptional regulation through protein lactylation. Emerging evidence suggests that the lactate–lactylation axis contributes to the pathogenesis of female reproductive diseases, including endometriosis, endometrial cancer, polycystic ovary syndrome, and ovarian cancer.

**Main body:**

This review summarizes current evidence on lactate production, lactate transport, and histone/non‐histone lactylation in female reproductive diseases. Available studies indicate that aberrant lactate accumulation and lactylation may influence chromatin accessibility, transcription factor activity, immune remodeling, ferroptosis resistance, steroidogenic dysfunction, and DNA damage repair. These processes may contribute to disease progression and may provide candidate biomarkers or therapeutic targets, including lactylation‐related enzymes, lactate transporters, glycolytic regulators, and lactate‐depleting strategies. However, the current evidence base remains dominated by cell and animal studies, and major barriers persist, including incomplete identification of bona fide reader proteins, insufficient standardization of site‐specific lactylation detection, limited validation in human cohorts, and uncertainty regarding disease specificity.

**Conclusion:**

The lactate–lactylation axis provides a useful framework for understanding metabolic–epigenetic coupling in female reproductive diseases and may inform future biomarker development, patient stratification, and mechanism‐based therapeutic strategies.

## Graphical abstract

1. Lactate‐derived histone lactylation links glycolytic reprogramming to epigenetic regulation in female reproductive diseases.

2. In endometriosis, lactylation may promote inflammatory remodelling, immune dysregulation and ferroptosis resistance.

3. In PCOS, lactylation connects metabolic disturbance to granulosa‐cell dysfunction and hyperandrogenism.

4. In endometrial and ovarian cancers, lactylation supports tumour progression, DNA repair, immune suppression and therapy resistance.

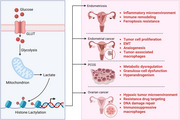

## 1 INTRODUCTION

The Warburg effect has long served as a central framework for understanding the metabolism of proliferating cells. This paradigm holds that, even under oxygen‐replete conditions, tumour cells preferentially convert glucose to lactate, bypassing mitochondrial oxidative phosphorylation. Historically, this phenomenon has been interpreted as a necessary adaptation to meet the biosynthetic demands of rapidly dividing cells, facilitating the generation of essential macromolecules such as nucleotides, amino acids and lipids. However, this biosynthetic framework does not fully account for the marked accumulation of intracellular and extracellular lactate observed in highly glycolytic cells.^1^, ^2^

In 2019, Zhang et al. identified histone lactylation as a previously unrecognised link between cellular metabolism and gene regulation.^3^ They showed that lactate is not merely a metabolic byproduct but can also serve as a metabolically derived donor for lysine lactylation (Kla). This post‐translational modification (PTM), called histone Kla, shares chemical similarities with acetylation. However, the unique steric structure and hydrophobicity of the lactyl group confer distinct physicochemical properties on chromatin. Importantly, lactylation levels appear to correlate with intracellular lactate availability and glycolytic activity, linking the cellular metabolic state directly to the transcriptional program. This novel PTM involves the enzymatic and covalent attachment of a lactyl group derived from lactate to lysine residues on proteins such as histones.^4^ Specifically, histone H3 lysine 18 lactylation (H3K18la) has been shown to directly activate transcriptional programs associated with macrophage M2 polarisation and glucose metabolism, thereby linking the cellular metabolic state to specific gene expression patterns and functions.^3^

Female reproductive disorders impose a substantial burden on fertility, quality of life and long‐term health. However, associated disorders – including endometriosis (EM), endometrial cancer, polycystic ovary syndrome (PCOS) and ovarian cancer – not only severely compromise patients’ fertility and quality of life but also represent a substantial global health burden.^5^, ^6^, ^7^, ^8^ The aetiology of these diseases is complex and multifactorial, involving dysregulation across genetic, endocrine and immunological systems, as well as metabolic imbalances within the local microenvironment.^9^, ^10^, ^11^ In recent years, the role of cellular metabolites and their derived novel PTMs in disease pathogenesis has garnered increasing attention. Notably, lactate metabolism and lactate‐mediated lactylation provide a potentially informative framework for understanding pathological mechanisms in female reproductive disorders.^12^

In the female reproductive system, lactate metabolism and lactylation are likely to be functionally linked. Reproductive tissues, such as the endometrium and ovaries, undergo cyclic, profound proliferation, differentiation and tissue remodelling. These dynamic processes are associated with significant shifts in energy metabolism. In the aforementioned diseases, metabolic reprogramming emerges as a shared and central pathophysiological hallmark.^13^, ^14^ Endometrial cells within endometriotic lesions exhibit enhanced glycolytic activity and invasive potential.^15^ As prototypical malignancies, endometrial and ovarian cancers are characterised by a pronounced Warburg effect, leading to substantial lactate accumulation within the tumour microenvironment.^16^, ^17^ PCOS is frequently associated with systemic and intra‐ovarian insulin resistance, as well as metabolic dysregulation.^18^ Under these pathological conditions, accumulated lactate may provide a permissive metabolic context for protein lactylation, which may in turn influence disease‐relevant cellular programs. Specifically, lactylation may affect transcription factors and histones involved in cell proliferation, invasion, adhesion, angiogenesis and immune regulation, thereby potentially contributing to disease progression.

This review summarises current evidence regarding the roles of lactate metabolism and lactate‐mediated lactylation in major female reproductive disorders. We outline the cascade from lactate generation and transport to lactylation and discuss how these processes may influence disease‐associated cell‐fate decisions and microenvironmental remodelling. By integrating current mechanistic and translational evidence, this review aims to clarify the potential relevance of the lactate–lactylation axis to biomarker development, disease stratification and mechanism‐informed therapeutic exploration.

## 2 MOLECULAR BASIS OF HISTONE LACTYLATION

### 2.1 The emergence of histone lactylation

Histone lactylation is a recently identified PTM in which a lactate‐derived lactyl group is covalently attached to the ε‐amino group of lysine residues.^3^ Compared with acetylation, the lactyl group is bulkier and contains an additional hydroxyl group, suggesting that lactylation may exert chromatin effects distinct from those of acetylation.

**FIGURE 1:**
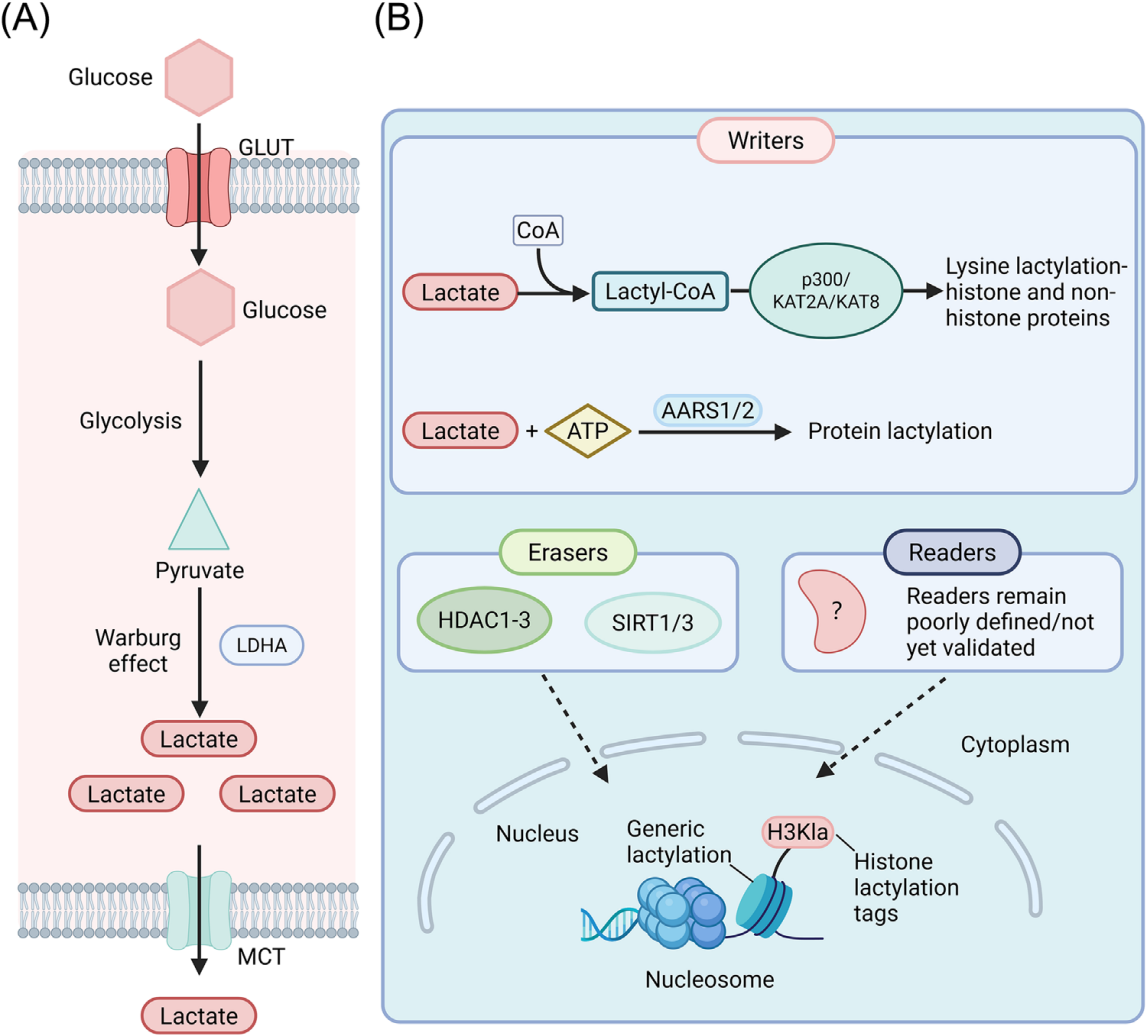
Metabolic origin and regulatory machinery of protein lactylation. (A) Under glycolytic conditions, glucose enters cells through glucose transporters and is converted to pyruvate through glycolysis. Pyruvate is then converted to lactate by LDHA, particularly under Warburg‐like metabolic conditions. Intracellular lactate may accumulate or be transported across the plasma membrane through MCTs, thereby influencing the lactate pool available for downstream lactylation. (B) Lactylation is regulated by writer, eraser and reader layers. In the canonical lactyl‐CoA‐dependent pathway, lactate‐derived lactyl‐CoA serves as a donor for p300/KAT2A/KAT8‐associated lysine lactylation of histone and non‐histone proteins. In parallel, AARS1/2 may mediate an ATP‐dependent, lactyl‐CoA‐independent form of protein lactylation. Lactylation marks may be removed by delactylases, including HDAC1–3 and SIRT1/3. Bona fide reader proteins that specifically recognise lactylated lysine residues remain poorly defined. Notation: Solid arrows denote experimentally supported or established pathways; dashed arrows denote proposed or incompletely defined mechanisms. *Abbreviations*: GLUT, glucose transporter; LDHA, lactate dehydrogenase A; MCT, monocarboxylate transporter; CoA, coenzyme A; ATP, adenosine triphosphate; AARS1/2, alanyl‐tRNA synthetase 1/2; KAT2A/KAT8, lysine acetyltransferase 2A/8; HDAC, histone deacetylase; SIRT, sirtuin; H3Kla, histone H3 lysine lactylation.

According to the classical Warburg effect, as shown in Figure 1A, rapidly proliferating cells, such as cancer cells and activated immune cells, preferentially convert glucose to pyruvate via glycolysis even under aerobic conditions. Pyruvate is then reduced to lactate by lactate dehydrogenase A (LDHA),^19^ a process that regenerates NAD^+^ to sustain a high glycolytic flux. When intracellular lactate accumulates, it may support protein lactylation through multiple emerging donor pathways, including lactyl‐CoA‐dependent mechanisms and alanyl‐tRNA synthetase 1/2 (AARS1/2)‐mediated lactyl‐AMP formation. Although the relative contribution of these routes may vary across biological contexts, current evidence suggests that lactyl donor generation is more diverse than previously appreciated.^3^, ^20^, ^21^

Monocarboxylate transporters (MCTs) are critical regulators of transmembrane lactate transport and are important determinants of intracellular lactate availability for protein lactylation. Their activity is an important determinant of intracellular lactate availability and may therefore influence protein lactylation levels.^22^ A recent study reported that monocarboxylate transporter 1 (MCT1) and monocarboxylate transporter 2 (MCT2) are expressed in adult neural stem cells, with MCT1 primarily mediating lactate efflux and MCT2 primarily mediating lactate influx.^23^ Conditional deletion of MCT1 resulted in intracellular lactate accumulation, increased histone lactylation and epigenetic activation of cell cycle‐related genes, thereby linking lactate transport to chromatin regulation. Similar observations have also been reported in tumour‐associated contexts, in which MCT1‐mediated lactate uptake was associated with increased histone lactylation in recipient cells, including immune cells exposed to tumour‐derived lactate, supporting the concept that MCT‐dependent lactate shuttling can couple metabolic crosstalk to lactylation‐dependent transcriptional remodelling.^24^, ^25^

This metabolic–epigenetic axis may also be relevant to the female reproductive system. In the ovary and endometrium, steroid hormones and their receptors have been shown to modulate local glucose metabolism and glycolytic programs, while MCT expression has been demonstrated in the female reproductive tract.^26^, ^27^, ^28^ These findings link lactate metabolism to reproductive endocrine regulation and support further investigation into the role of lactylation in physiological cycles and reproductive pathologies.

### 2.2 The regulatory machinery of lactylation: writers, erasers and readers

Lactylation is a reversible PTM that appears to arise through at least two mechanistically distinct routes: a canonical enzymatic pathway that uses an activated lactyl donor, most commonly presumed to be lactyl‐CoA, and a recently described ATP‐dependent, lactyl‐CoA‐independent pathway. In epigenetic terminology, ‘writers’ are enzymes that install lactyl marks onto lysine residues, ‘erasers’ are enzymes that remove these marks and ‘readers’ are proteins that recognise lactylated lysines and help translate this modification into downstream chromatin or transcriptional effects (Figure 1B).^3^, ^29^ In the canonical enzymatic pathway, several histone acetyltransferase‐family enzymes, including E1A‐binding protein p300 (p300), lysine acetyltransferase 2A (KAT2A) and lysine acetyltransferase 8 (KAT8), have been proposed to function as lactylation ‘writers’, by transferring a lactyl group from an activated donor, generally presumed to be l‐lactyl‐CoA, to lysine residues on histone and non‐histone substrates. However, the biosynthetic route leading to intracellular lactyl‐CoA remains incompletely defined. Current evidence suggests that this intermediate may not arise through a single dedicated pathway; rather, it may be generated under specific metabolic conditions through the promiscuous activity of acyl‐CoA synthetase family members, particularly short‐chain acyl‐CoA synthetases, although the relative contribution of individual enzymes is likely to be cell‐type and context dependent.^30^, ^31^ Thus, although lactyl‐CoA is widely invoked as the canonical donor for p300/KAT‐mediated lactylation, the quantitative abundance, subcellular compartmentalisation and disease‐specific regulation of lactyl‐CoA remain insufficiently resolved. A major recent advance was the identification of AARS1 and AARS2 as ATP‐dependent lactyltransferases that mediate a lactyl‐CoA‐independent form of Kla. In contrast to the canonical p300/KAT model, which relies on a pre‐activated acyl donor, the AARS1/2 system appears to use lactate and ATP directly, thereby bypassing the requirement for lactyl‐CoA. Mechanistically, this finding is important because it introduces a conceptually distinct route of lactylation control in which aminoacyl‐tRNA synthetase family members act as metabolic sensors that directly couple lactate availability to proteome‐wide lysine modification. This mechanism may be particularly relevant in cellular states marked by acute lactate accumulation, where rapid donor‐independent lactylation could occur even if lactyl‐CoA production is limited.^32^ At present, it remains unclear whether the AARS1/2‐dependent pathway and the p300/KAT‐dependent pathway operate in parallel, in different subcellular compartments or under distinct metabolic constraints. One plausible model is that the p300/KAT system preferentially regulates locus‐selective chromatin lactylation when activated donor pools are available, whereas AARS1/2 may support broader or more rapid proteome‐level lactylation under conditions of high lactate flux. Alternatively, the two systems may compete for overlapping substrates in a cell‐context‐dependent manner. Defining how these pathways cooperate, partition substrates or dominate in specific disease settings will be essential for interpreting lactylation as a mechanistic signal rather than a generic metabolic correlate. Regarding the removal of lactylation, several histone deacetylases (HDACs) have been verified to function as ‘erasers’ (or delactylases). These include Class I HDACs (specifically HDAC1–3) and Class III deacetylases (such as sirtuin 1 (SIRT1) and sirtuin 1 (SIRT3)). These enzymes catalyse the removal of lactyl groups, thereby enabling dynamic and reversible regulation of lactylation.^4^, ^33^, ^34^ Ultimately, the biological consequences of lactylation depend not only on its deposition and removal but also on the ability of downstream effector proteins to recognise and decode lactyl‐lysine marks. However, bona fide reader proteins with validated specificity for lactylated residues remain largely undefined. This gap is mechanistically important: while marks such as H3K18la are consistently associated with transcriptional activation, the absence of well‐validated reader proteins means that the direct molecular pathway linking lactylation to transcriptional initiation, chromatin remodelling or cofactor recruitment remains unresolved.

The biological relevance of lactylation extends beyond histones. In 2025, Liu et al. reported that follicle stimulating hormone (FSH) stimulation promotes lactylation of the transcription factor cAMP response element‐binding protein (CREB) at lysine 136 (K136la), thereby facilitating subsequent CREB activation.^35^ Additional studies have suggested that lactylation may also regulate other transcription factors, including YY1, where it has been linked to increased transcriptional activity in tumour‐related settings.^36^ Beyond transcription factors, lactylation has also been reported to regulate the function of other non‐histone proteins involved in post‐transcriptional control. For example, Fang et al.^37^ reported that the acyltransferase KAT2A mediates regulator of chromosome condensation 2 (RCC2) lactylation at K124, thereby promoting the recruitment of free SERBP1 by RCC2 and stabilising MAD2L1 mRNA. Collectively, these findings indicate that lactylation is not restricted to histones but also affects diverse non‐histone proteins, including transcription factors and regulatory proteins involved in RNA stability or signal transduction. In currently reported examples, transcription factor lactylation is most often associated with enhanced transactivation rather than repression, although its functional consequence is likely to remain context dependent.

Currently, H3K18la has become one of the most frequently discussed lactylation marks.^38^ Existing evidence indicates that lactylation is not restricted to a single histone site. Other histone sites, including H3K9la, H4K12la and H3K56la, have also been implicated in distinct regulatory programs. Among them, H3K9la has been linked to chromatin‐associated transcriptional regulation and, in ovarian cancer, to activation of homologous recombination or DNA repair‐related gene programs,^39^, ^40^ whereas H4K12la has been associated with super‐enhancer activity and treatment resistance.^41^ H3K56la has also been implicated in stemness‐related transcriptional regulation, although current evidence for this mainly comes from non‐gynaecologic models.^42^ Together, these findings suggest that the functional consequences of lactylation are site‐ and substrate‐dependent and that a broader view of lactylation beyond H3K18la is necessary to understand its role in female reproductive diseases.

As a potential interface between metabolic reprogramming and signalling regulation, lactylation has been increasingly implicated in the pathogenesis of several female reproductive diseases. Specifically, in EM, infiltrating immune cells and aberrant endometrial cells within lesions generate excessive lactate via hyperactive glycolysis, thereby shaping a unique acidic microenvironment.^43^, ^44^ Accumulated lactate has been reported to induce lactylation of key transcription factors, such as hypoxia‐inducible factor 1α (HIF‐1α) and signal transducer and activator of transcription 1 (STAT1). This modification thereby potentiates the invasive, adhesive and survival capabilities of the lesional cells.^45^, ^46^ In endometrial and ovarian cancers, the Warburg effect leads to excessive lactate accumulation within the tumour microenvironment.^44^ This lactate may provide a substrate context that favours histone lactylation at H3K18. Consequently, H3K18la activates gene expression programs associated with epithelial–mesenchymal transition, angiogenesis and cell proliferation, thereby supporting gene‐expression programs associated with malignant progression.^47^, ^48^, ^49^ PCOS, metabolic dysregulation in ovarian granulosa cells (GCs) and insulin resistance may collectively contribute to elevated local lactate levels.^18^, ^50^ Accumulated lactate has been proposed to influence granulosa‐cell function through protein lactylation; however, the precise downstream targets and regulatory mechanisms remain incompletely defined. Current evidence suggests that lactylation may affect selected transcriptional regulators and other non‐histone proteins involved in metabolic and endocrine responses, thereby potentially contributing to abnormal folliculogenesis and ovulatory dysfunction.^51^

## 3 MECHANISMS OF LACTYLATION IN FEMALE REPRODUCTIVE DISEASES

### 3.1 Endometriosis

EM is a chronic gynaecological disorder characterised by the presence of endometrial‐like tissue outside the uterine cavity, affecting approximately 10% of reproductive‐age women globally.^52^ The side effects of hormonal therapies and high post‐surgical recurrence rates have long hampered the clinical management of EM. Accordingly, a better understanding of its molecular basis may facilitate the development of more effective therapeutic strategies. Although histologically benign, ectopic endometrial tissue exhibits ‘tumour‐like’ molecular behaviours, characterised by resistance to apoptosis, induction of angiogenesis, invasiveness and profound metabolic reprogramming.^53^, ^54^

The lactate‐rich environment within endometriotic lesions was initially interpreted mainly as a consequence of metabolic reprogramming. However, the discovery of histone lactylation has suggested that lactate may also function as a signalling metabolite linking metabolism to transcriptional regulation.^3^ In the endometriotic microenvironment, hypoxia and inflammatory stimuli drive substantial metabolic reprogramming, resulting in increased lactate production.^55^, ^56^ A fundamental prerequisite for lactylation is a high intracellular lactate pool. Consistent evidence indicates that ectopic endometrial tissue exhibits enhanced aerobic glycolysis compared with eutopic endometrium, supporting a metabolically rewired state in EM. This metabolic shift provides ATP and essential carbon skeletons for biosynthesis, fuelling proliferating cells. LDHA, a key rate‐limiting enzyme in the glycolytic pathway, catalyses the conversion of pyruvate into lactate. In ectopic lesions, LDHA expression is significantly elevated and is associated with increased glycolytic activity and a lactate‐rich microenvironment. This LDHA up‐regulation is therefore likely to contribute to lactate accumulation and to create a metabolic context permissive for lactylation.^57^, ^58^ To sustain high‐flux glycolysis, ectopic endometrial stromal cells (EESCs) substantially increase glucose uptake by up‐regulating glucose transporter 1 (GLUT1).

**FIGURE 2:**
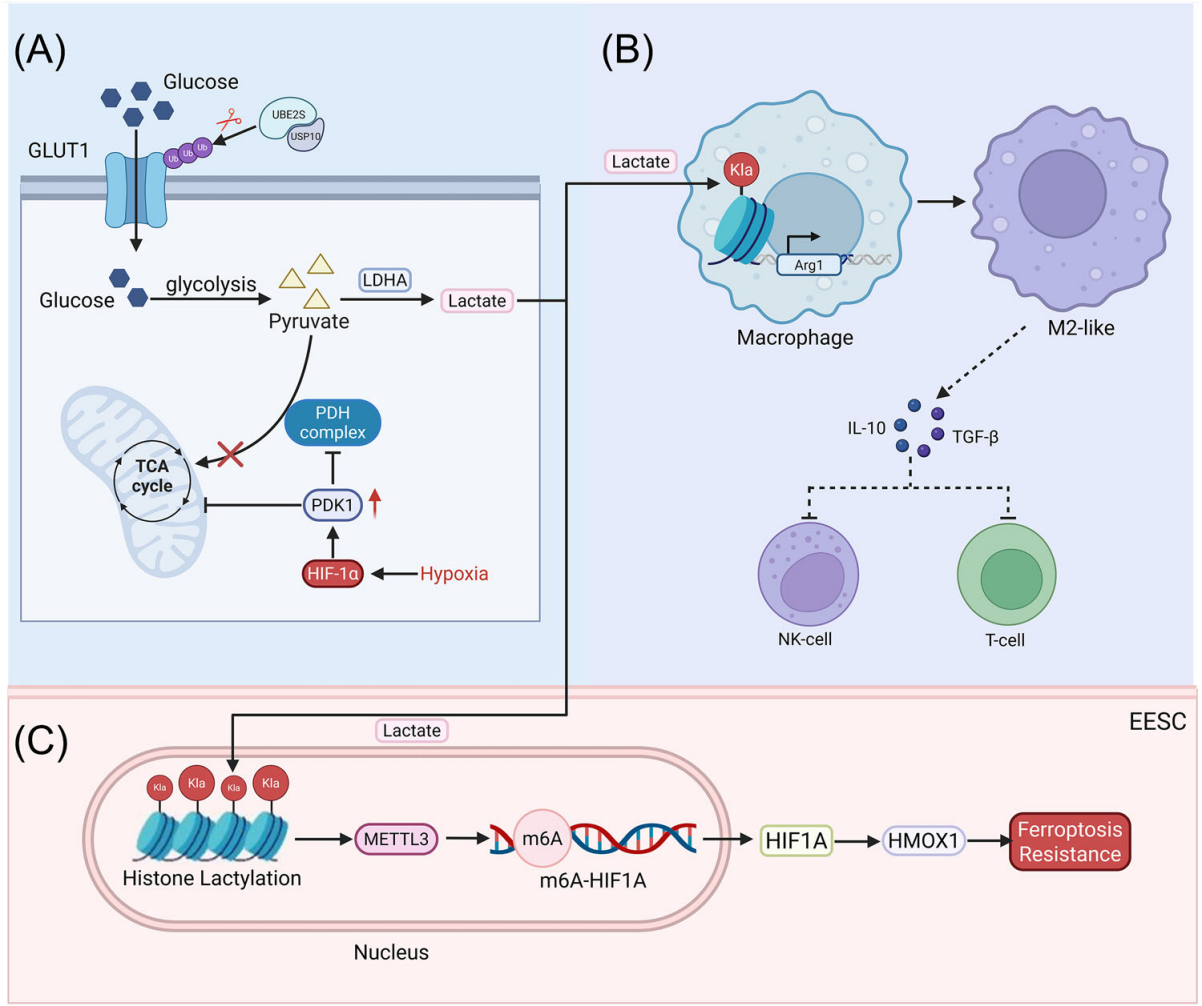
Proposed roles of lactate‐associated lactylation in ferroptosis resistance and immune remodelling in endometriosis. (A) In ectopic endometrial stromal cells (EESCs), enhanced glucose uptake and glycolytic flux increase lactate production. UBE2S/USP10‐mediated stabilisation of GLUT1 may reinforce glucose uptake, whereas hypoxia‐induced HIF‐1α up‐regulates PDK1. PDK1 inhibits the PDH complex, restricts mitochondrial pyruvate entry into the TCA cycle and favours pyruvate‐to‐lactate conversion through LDHA. Together, these events create a lactate‐rich intracellular and extracellular microenvironment. (B) Macrophages may take up lactate released from EESCs in the peritoneal microenvironment. Lactate‐associated histone lactylation is linked to Arg1 expression and M2‐like macrophage polarisation. M2‐like macrophages may secrete IL‐10 and TGF‐β, thereby suppressing NK‐cell and T‐cell cytotoxicity and supporting immune escape of ectopic lesions. (C) In EESCs, lactate accumulation is associated with increased histone lactylation, particularly H3K18la, which promotes METTL3 expression. METTL3 enhances HIF1A mRNA stability or translation through m6A‐related mechanisms, leading to HIF1A/HMOX1 activation and ferroptosis resistance in iron‐rich endometriotic lesions. Notation: Solid arrows denote experimentally supported or established pathways; dashed arrows denote proposed or incompletely defined mechanisms. Red upward arrows indicate increased expression, abundance, activity or modification level. Blunt‐ended lines indicate inhibition. Red ‘Kla’ circles indicate lysine lactylation. The scissors symbol in panel A denotes USP10‐mediated deubiquitination. *Abbreviations*: EESCs, ectopic endometrial stromal cells; GLUT1, glucose transporter 1; UBE2S, ubiquitin‐conjugating enzyme E2S; USP10, ubiquitin‐specific peptidase 10; LDHA, lactate dehydrogenase A; PDH, pyruvate dehydrogenase; PDK1, pyruvate dehydrogenase kinase 1; TCA, tricarboxylic acid; HIF‐1α/HIF1A, hypoxia‐inducible factor 1 alpha; Kla, lysine lactylation; Arg1, arginase 1; M2‐like, alternatively activated macrophage‐like phenotype; IL‐10, interleukin‐10; TGF‐β, transforming growth factor beta; NK cell, natural killer cell; METTL3, methyltransferase‐like 3; m6A, N6‐methyladenosine; HMOX1, heme oxygenase 1.

Endometriotic lesions are embedded within the complex microenvironment of the peritoneum and visceral organs, where compromised local perfusion and inflammatory stress can create a hypoxic niche. Under these conditions, HIF‐1α is stabilised and acts as a central transcriptional regulator of glycolytic reprogramming. HIF‐1α can up‐regulate key glycolysis‐related genes, including LDHA, SLC2A1 encoding GLUT1 and pyruvate dehydrogenase kinase 1 (PDK1), thereby increasing glucose uptake, promoting pyruvate‐to‐lactate conversion and limiting mitochondrial pyruvate oxidation. Mechanistically, PDK1 phosphorylates and inhibits the pyruvate dehydrogenase (PDH) complex, restricting pyruvate entry into the tricarboxylic acid cycle and redirecting carbon flux towards lactate production.^54^, ^56^ In parallel, Wang et al.^59^ recently showed that the ubiquitin‐conjugating enzyme ubiquitin‐conjugating enzyme E2S (UBE2S) recruits the deubiquitinase ubiquitin‐specific peptidase 10 (USP10) to remove K48‐linked ubiquitin chains from GLUT1, thereby stabilising GLUT1 and further enhancing glucose uptake and glycolytic flux. As illustrated in Figure 2A, hypoxia‐induced HIF‐1α/PDK1 signalling, PDH inhibition, LDHA‐mediated lactate production and UBE2S/USP10‐dependent stabilisation of GLUT1 together shift EESC metabolism towards lactate accumulation. This regulatory axis may contribute to sustained lactate production within EM lesions.^56^, ^60^ Moreover, the long non‐coding RNA (lncRNA) H19 is aberrantly up‐regulated in ectopic endometrial tissues. Functional studies indicate that up‐regulated H19 promotes cell proliferation while enhancing aerobic glycolysis levels, lactate production and histone lactylation in EM, thereby linking lncRNA dysregulation to lactate‐dependent epigenetic remodelling.^53^ In parallel, the lesions actively affect surrounding immune and stromal cells. Macrophages are the most abundant immune cell population in peritoneal fluid. Within the peritoneal microenvironment, lactate may function as an immunomodulatory metabolite. This suggests that lactate in the peritoneal microenvironment may influence macrophage polarisation through histone lactylation. In macrophage systems, lactate‐driven histone lactylation has been linked to the induction of reparative genes such as arginase 1 (Arg1) and to a shift towards an M2‐like phenotype; in EM, this mechanism is biologically plausible but remains less directly established than stromal‐cell‐intrinsic lactylation pathways.^55^, ^61^, ^62^ Such M2‐skewed macrophages are expected to produce immunoregulatory cytokines such as interleukin‐10 (IL‐10) and transforming growth factor‐β (TGF‐β,) which may dampen natural killer (NK) and T cell cytotoxicity and thereby favour immune escape of ectopic lesions.^63^ This immune‐regulatory arm is depicted in Figure 2B.

Additionally, EM lesions are characterised by cyclic bleeding and extreme local iron overload, conditions that theoretically predispose cells to ferroptosis. Nevertheless, EESCs appear to display relative resistance to ferroptosis. In 2025, Liang et al.^54^ showed that glycolysis‐driven lactate accumulation increases H3K18la, thereby up‐regulating the N6‐methyladenosine (m6A) methyltransferase methyltransferase‐like 3 (METTL3). In turn, METTL3 enhances HIF‐1α expression through m6A‐dependent stabilisation and/or translation of its mRNA, leading to activation of the downstream effector heme oxygenase‐1 (HMOX1) (Figure 2C). In this model, HMOX1 induction was associated with ferroptotic resistance, suggesting that the H3K18la–METTL3–HIF1A/HMOX1 axis may help ectopic lesions survive in an iron‐rich oxidative microenvironment. Notably, combined treatment with the glycolysis inhibitor 2‐DG and the ferroptosis inducer erastin reduced lesion volume in vivo, supporting further preclinical evaluation of this combinatorial strategy. The H3K18la–METTL3–HIF‐1α–HMOX1 axis currently represents one of the more mechanistically developed models in EM; it remains unclear whether H3K18la acts as a direct instructive signal or as a broader chromatin correlate of glycolytic rewiring, particularly because the reader proteins that would decode this mark in ectopic stromal cells have not yet been identified.

### 3.2 Endometrial cancer

Endometrial cancer (EC), which arises from the endometrial epithelium, is the most common gynaecologic malignancy in developed nations.^64^ Its incidence has risen substantially over the past three decades, a trend that has been associated with increasing obesity, population aging and the growing burden of metabolic syndrome.^65^ Clinically, most patients with early‐stage EC achieve favourable outcomes with surgery‐based management, typically total hysterectomy with bilateral salpingo‐oophorectomy; adjuvant therapy is then individualised according to stage, histology, risk factors and increasingly, molecular classification. By contrast, patients with advanced or recurrent EC, particularly those with aggressive histologies such as serous or clear cell carcinoma, continue to experience poorer outcomes. Although platinum‐taxane chemotherapy remains a key backbone of treatment, current first‐line management increasingly incorporates immune checkpoint inhibitors and therapeutic resistance remains a major clinical challenge.^66^

**FIGURE 3:**
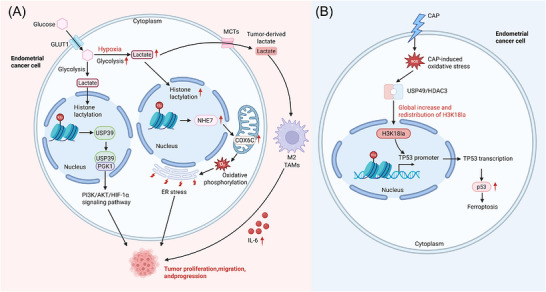
Context‐dependent roles of histone lactylation in endometrial cancer and CAP‐associated ferroptosis. (A) Under basal malignant conditions, endometrial cancer cells exhibit enhanced glucose uptake, hypoxia‐associated glycolysis and lactate accumulation. Increased histone lactylation, particularly H3K18la, is linked to activation of the USP39–PGK1–PI3K/AKT/HIF‐1α signalling axis, thereby promoting tumour proliferation, migration and progression. Histone lactylation may also activate NHE7 and COX6C‐associated mitochondrial or endoplasmic‐reticulum stress‐adaptation pathways. Tumour‐derived lactate exported through MCTs may further promote M2‐like polarisation of TAMs, which secrete IL‐6 and reinforce a feed‐forward tumour‐promoting microenvironment. (B) Under CAP‐induced oxidative stress, the USP49/HDAC3 axis is associated with global elevation and redistribution of H3K18la. In this stress context, H3K18la may become enriched at the TP53 promoter, promoting TP53 transcription, p53 induction and ferroptosis. Thus, H3K18la may support tumour‐promoting transcriptional programs under basal malignant conditions but contribute to tumour‐suppressive ferroptotic responses under intense oxidative stress. Notation: Solid arrows denote experimentally supported or established pathways. Red upward arrows indicate increased expression, abundance, activity or modification level. Red ‘Kla’ circles indicate lysine lactylation. *Abbreviations*: EC, endometrial cancer; GLUT1, glucose transporter 1; MCTs, monocarboxylate transporters; H3K18la, histone H3 lysine 18 lactylation; USP39, ubiquitin‐specific peptidase 39; PGK1, phosphoglycerate kinase 1; PI3K, phosphoinositide 3‐kinase; AKT, protein kinase B; HIF‐1α, hypoxia‐inducible factor 1 alpha; NHE7, sodium/hydrogen exchanger 7; COX6C, cytochrome c oxidase subunit 6C; ER, endoplasmic reticulum; TAMs, tumour‐associated macrophages; IL‐6, interleukin‐6; CAP, cold atmospheric plasma; ROS, reactive oxygen species; USP49, ubiquitin‐specific peptidase 49; HDAC3, histone deacetylase 3; TP53, tumour protein p53 gene; p53, tumour suppressor protein p53.

Lactate, generated and secreted into the microenvironment by endometrial cancer cells through aerobic glycolysis, may function not only as a substrate for lactylation but also as a signalling metabolite. Liu et al.^67^ demonstrated that EC‐derived lactate induces M2 polarisation of tumour‐associated macrophages (TAMs). As shown on the right side of Figure 3A, these lactate‐induced M2 TAMs, in turn, engage an IL‐6‐centred signalling circuit that feeds back to tumour cells and supports endometrial cancer progression. More broadly, lactylation can also regulate the activity of key transcription factors. For example, in non‐endometrial systems, YY1 lactylation enhances fibroblast growth factor (FGF2) transcription, supporting the broader concept that lactylation may influence angiogenic gene programs; however, an equivalent mechanism has not yet been demonstrated in EC.^68^ At the metabolic level, enhanced aerobic glycolysis in endometrial carcinoma promotes lactate accumulation, which is accompanied by increased histone lactylation.^69^, ^70^ Among the lactylation marks examined, H3K18la appears to be particularly elevated in EC and is broadly recognised as an active chromatin mark.^38^ H3K18la–USP39 axis, recent studies suggest that histone lactylation in endometrial cancer may contribute to broader stress adaptation programs. A 2026 study reported that hypoxia‐driven glycolysis promotes histone lactylation and activates NHE7, thereby facilitating endometrial cancer progression through COX6C‐mediated endoplasmic reticulum homeostasis.^71^ In contrast, cold atmospheric plasma (CAP) is an emerging physical therapeutic modality. In a recent study, CAP‐induced oxidative stress triggered ferroptosis in EC cells through the ubiquitin‐specific peptidase 49 (USP49)/histone deacetylase 3 (HDAC3) axis, accompanied by global up‐regulation of H3K18la. Under these stress conditions, H3K18la appears to be redistributed towards the tumor protein p53 gene (TP53) promoter, thereby enhancing p53 transcription and triggering ferroptosis^72^ (Figure 3B). These findings suggest that the function of H3K18la is highly context dependent: in the basal malignant state, it promotes activation of oncogenic programs such as USP39, whereas under intense exogenous stress, chromatin remodelling may redirect H3K18la towards tumour‐suppressive programs, such as TP53 activation. This context dependence provides a potential rationale for therapeutically exploiting lactylation‐linked epigenetic reprogramming in EC.

### 3.3 Polycystic ovary syndrome

PCOS is a highly heterogeneous reproductive endocrine disorder affecting approximately 4–21% of women of reproductive age worldwide.^73^ Our understanding of its pathogenesis has evolved from a hypothesis of singular hormonal dysregulation to a complex, multidimensional ‘immune–metabolic–epigenetic’ network.^18^ Within this pathological context, ovarian GCs exhibit metabolic dysfunction, including dysregulated coordination between glycolysis and mitochondrial activity. Current evidence suggests that glycolytic pathways are reprogrammed in PCOS‐derived GCs, although the direction and magnitude of this change may vary across models, follicular stages and biological compartments.^74^, ^75^, ^76^, ^77^ Accordingly, lactate handling in PCOS should not be viewed as uniformly increased or decreased, but rather as context dependent.

**FIGURE 4:**
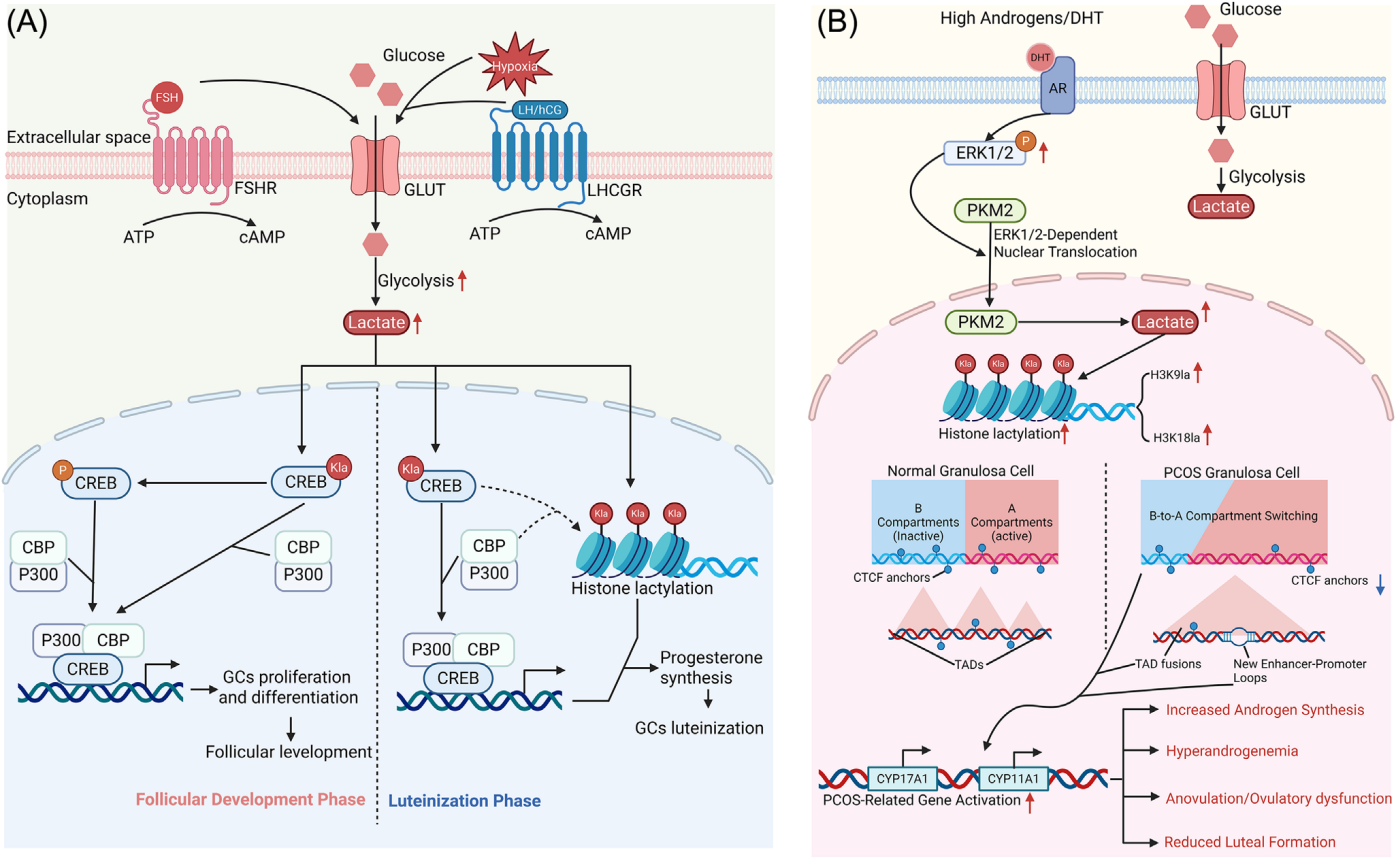
Lactate‐linked transcriptional and chromatin remodelling mechanisms in granulosa cells during physiological follicular development and PCOS. (A) Under physiological stimulation, FSH activates FSHR and the cAMP‐dependent signalling pathway in granulosa cells. FSH‐driven glucose uptake and glycolysis increase lactate production, which supports CREB lactylation at lysine 136. CREB K136 lactylation facilitates CBP/p300 recruitment and promotes gene expression programs required for granulosa‐cell proliferation, differentiation and follicular development. During luteinisation, LH/hCG signalling through LHCGR and hypoxia‐associated glycolytic activation may further increase lactate production and histone lactylation, supporting progesterone synthesis and granulosa‐cell luteinisation. (B) In PCOS granulosa cells, high androgens/DHT activate AR‐related signalling and ERK1/2 phosphorylation, promoting PKM2 nuclear translocation. Nuclear PKM2, together with increased glycolytic lactate production, is associated with increased H3K9la and H3K18la. These histone lactylation marks may remodel three‐dimensional genome organisation, including B‐to‐A compartment switching, CTCF‐anchor disruption, TAD fusion and the formation of new enhancer–promoter loops. These chromatin changes activate steroidogenic genes such as CYP17A1 and CYP11A1, contributing to increased androgen synthesis, hyperandrogenaemia, anovulation or ovulatory dysfunction and impaired luteal formation. Notation: Solid arrows denote experimentally supported or established pathways. Red upward arrows indicate increased expression, abundance, activity or modification level. Red ‘Kla’ circles indicate lysine lactylation; ‘P’ indicates phosphorylation. *Abbreviations*: FSH, follicle‐stimulating hormone; FSHR, FSH receptor; LH, luteinising hormone; hCG, human chorionic gonadotropin; LHCGR, LH/hCG receptor; cAMP, cyclic adenosine monophosphate; CREB, cAMP response element‐binding protein; K136la, lysine 136 lactylation; CBP, CREB‐binding protein; p300, E1A‐binding protein p300; GCs, granulosa cells; PCOS, polycystic ovary syndrome; DHT, dihydrotestosterone; AR, androgen receptor; ERK1/2, extracellular signal‐regulated kinase 1/2; PKM2, pyruvate kinase M2; H3K9la, histone H3 lysine 9 lactylation; H3K18la, histone H3 lysine 18 lactylation; CTCF, CCCTC‐binding factor; TADs, topologically associating domains; CYP17A1, cytochrome P450 family 17 subfamily A member 1; CYP11A1, cytochrome P450 family 11 subfamily A member 1.

Beyond its conventional role as a metabolic byproduct, lactate can also act as a signalling metabolite by modulating the lactylation of histone and non‐histone proteins, thereby influencing gene expression programs relevant to folliculogenesis. Mechanistic support for this concept first comes from physiological granulosa‐cell models. As illustrated in Figure 4A, FSH‐driven glycolysis provides lactate for CREB K136 lactylation, which facilitates CREB‐binding protein (CBP)/p300 recruitment and supports granulosa‐cell proliferation, differentiation and follicular development.^35^ The luteinisation branch of Figure 4A further suggests that hypoxia and luteinizing hormone/human chorionic gonadotropin (LH/hCG)‐associated glycolytic activation may increase lactate production and histone lactylation, thereby supporting progesterone synthesis and granulosa‐cell luteinisation.^78^ Although this study was not performed specifically in PCOS, it offers a plausible framework for understanding how lactate‐sensitive transcriptional regulation could affect follicular development in PCOS. More direct disease‐specific evidence has emerged from recent studies of metabolic–epigenetic crosstalk in PCOS GCs. Wang et al.^79^ recently showed that androgen excess drives solute carrier family 1 member 5 (SLC1A5)‐dependent glutamine reprogramming in GCs. The resulting accumulation of glutamine‐derived α‐ketoglutarate enhances histone deacetylase 5 (HDAC5) expression and suppresses histone acetylation, particularly H3K14ac and H3K56ac, thereby down‐regulating folliculogenesis‐related genes such as cytochrome P450 family 19 subfamily A member 1 (CYP19A1) and further aggravating androgen imbalance. In parallel, Yu et al.^80^ provided the first systematic evidence of the epigenetic function of the glycolytic enzyme pyruvate kinase M2 (PKM2) in PCOS. As summarised in Figure 4B, a significant increase in the nuclear translocation of PKM2 within PCOS GCs. Once in the nucleus, nuclear PKM2 has been proposed to facilitate histone lactylation at H3K9 and H3K18 (H3K9la, H3K18la). These modifications directly remodel the 3D genome architecture, triggering chromatin compartment switching and the reorganisation of topologically associating domains (TADs). Consequently, the promoter regions of key steroidogenic genes, such as cytochrome P450 family 17 subfamily A member 1 (CYP17A1), become aberrantly accessible, driving excessive androgen synthesis. These findings expand the current understanding of hyperandrogenism by suggesting that metabolic byproducts may also participate in regulating steroidogenic genes. A contemporaneous lactyl‐proteomic analysis of GCs from patients with PCOS further revealed widespread alterations in lactylated proteins and lactylation sites in the PCOS group. Although this study was not limited to histones, it nevertheless demonstrated systematic lactylation remodelling in PCOS GCs. The authors also explicitly noted, however, that the precise functional significance and translational potential of lactylation in PCOS still require more in‐depth mechanistic validation.^81^

Besides, histone lactylation in PCOS does not appear to be restricted to the ovary. Recent evidence from the endometrium indicates that excessive histone lactylation is associated with estrogen receptor alpha (ERα) up‐regulation, aberrant oestrogen‐responsive transcription and impaired endometrial receptivity. In contrast, inhibition of lactate production or lactylation can improve implantation in a PCOS mouse model.^82^ Taken together, these findings suggest that histone lactylation is emerging as a plausible epigenetic mediator of both ovarian and endometrial dysfunction in PCOS, linking metabolic disturbance to abnormal folliculogenesis, hyperandrogenism and impaired reproductive competence.

### 3.4 Ovarian cancer

**FIGURE 5:**
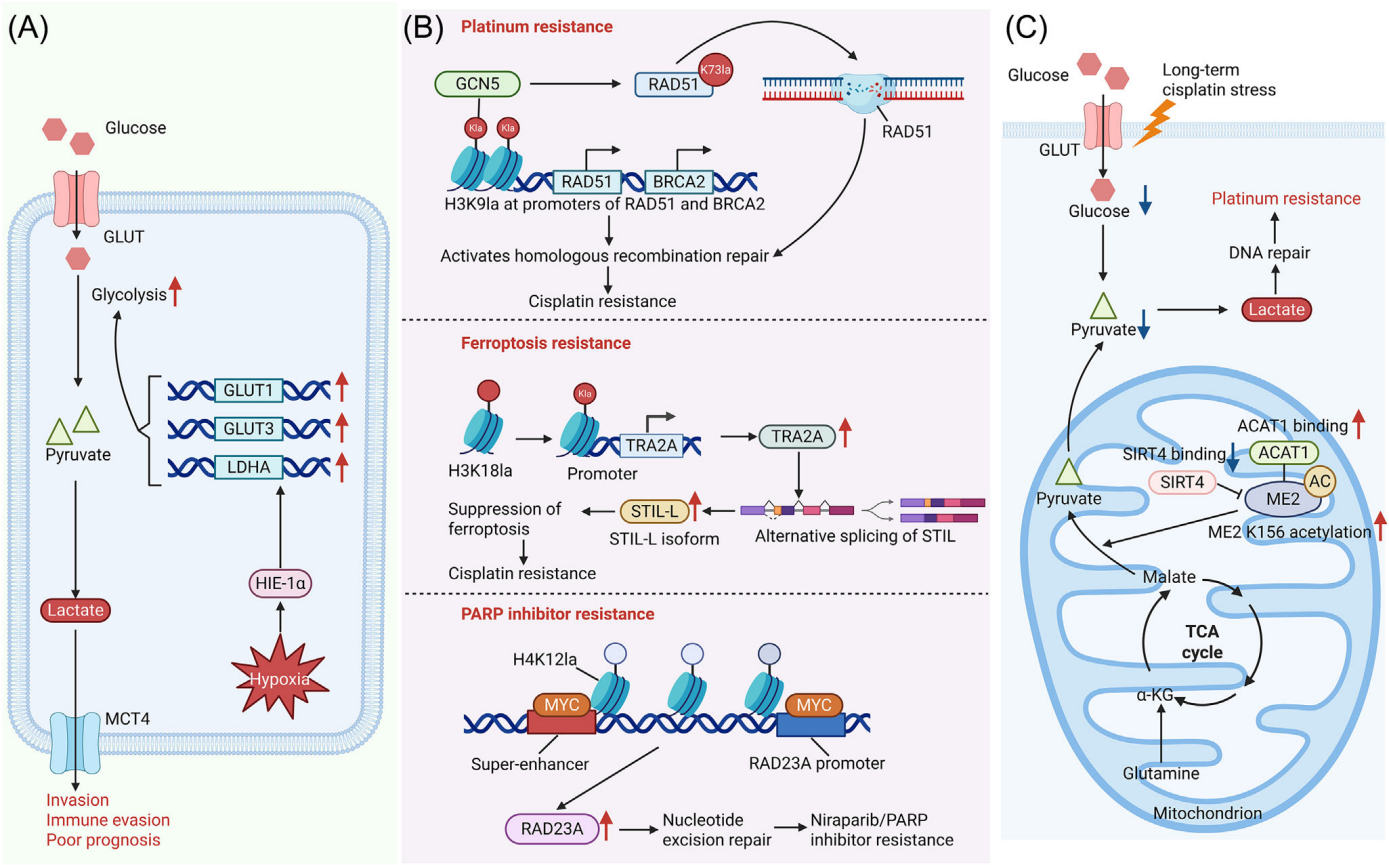
Lactylation‐related mechanisms contributing to metabolic adaptation, DNA repair and therapeutic resistance in ovarian cancer. (A) In ovarian cancer cells, hypoxia stabilises HIF‐1α and promotes glycolytic reprogramming, including increased expression of GLUT1/GLUT3 and LDHA. Enhanced glycolysis increases lactate production, while MCT4‐mediated lactate export contributes to extracellular acidification, invasion, immune evasion and poor prognosis. (B) Lactylation is implicated in multiple therapy‐resistance modules. In platinum‐resistant ovarian cancer, GCN5‐mediated H3K9la at the promoters of RAD51 and BRCA2 activates homologous recombination repair, while non‐histone RAD51 K73 lactylation enhances RAD51 function at DNA damage sites, jointly promoting cisplatin resistance. H3K18la is also linked to TRA2A activation, STIL alternative splicing, STIL‐L isoform generation, ferroptosis suppression and cisplatin resistance. In PARP inhibitor‐resistant cells, H4K12la activates a super‐enhancer upstream of RAD23A and recruits MYC, thereby increasing RAD23A expression, enhancing nucleotide excision repair and contributing to niraparib/PARP inhibitor resistance. (C) Under long‐term cisplatin stress and glucose‐limited conditions, ACAT1 binding and ME2 K156 acetylation support glutamine anaplerosis, TCA‐cycle flux, malate‐to‐pyruvate conversion and sustained lactate production. This metabolic compensation may preserve lactate availability for downstream lactylation‐associated DNA repair and platinum resistance, whereas SIRT4 may counteract ME2 acetylation. Notation: Solid arrows denote experimentally supported or established pathways; red upward arrows indicate increased expression, abundance, activity or modification level, whereas blue downward arrows indicate decreased expression, abundance, activity or metabolic flux. Blunt‐ended lines indicate inhibition. Red ‘Kla’ circles indicate lysine lactylation; ‘P’ indicates phosphorylation; ‘Ac’ indicates acetylation. HGSOC, high‐grade serous ovarian carcinoma; GLUT1/3, glucose transporter 1/3; HIF‐1α, hypoxia‐inducible factor 1 alpha; LDHA, lactate dehydrogenase A; MCT4, monocarboxylate transporter 4; GCN5, general control nonderepressible 5/KAT2A; H3K9la, histone H3 lysine 9 lactylation; H3K18la, histone H3 lysine 18 lactylation; H4K12la, histone H4 lysine 12 lactylation; RAD51, RAD51 recombinase; BRCA2, breast cancer gene 2; HR, homologous recombination; TRA2A, transformer‐2 protein homolog alpha; STIL, SCL/TAL1‐interrupting locus; STIL‐L, long STIL isoform; PARP, poly(ADP‐ribose) polymerase; RAD23A, RAD23 homolog A; MYC, MYC proto‐oncogene; NER, nucleotide excision repair; ACAT1, acetyl‐CoA acetyltransferase 1; ME2, malic enzyme 2; K156ac, lysine 156 acetylation; SIRT4, sirtuin 4; α‐KG, α‐ketoglutarate; TCA, tricarboxylic acid.

Ovarian cancer, particularly high‐grade serous ovarian carcinoma (HGSOC), is characterised by extensive metabolic reprogramming.^83^ Its distinct pattern of transcoelomic dissemination, coupled with a malignant ascites milieu enriched in lipids and often accompanied by elevated lactate, creates a microenvironment conducive to metabolic adaptation.^84^ In ovarian cancer cells, enhanced aerobic glycolysis promotes intracellular lactate accumulation, which may increase substrate availability for histone lactylation.^19^ Hypoxia is a major metabolic stressor in ovarian cancer and HIF‐1α stabilisation under hypoxic conditions promote the expression of glycolysis‐associated genes, including GLUT1, GLUT3 and LDHA.^85^, ^86^ In parallel, monocarboxylate transporter 4 (MCT4)‐mediated lactate export contributes to extracellular acidification and to an acidic microenvironment associated with invasion, immune evasion and poor prognosis.^87^, ^88^, ^89^ This hypoxia‐driven metabolic background is summarised in Figure 5A.

Against this metabolic background, recent studies on ovarian cancer have extended the functional relevance of lactylation to therapeutic resistance. In platinum‐resistant ovarian cancer, elevated histone lactylation – particularly H3K9la – and RAD51 recombinase (RAD51) K73 lactylation enhance homologous recombination repair and support cisplatin resistance. As illustrated in the platinum resistance area of Figure 5B, general control nonderepressible 5 protein (GCN5) mediates H3K9 lactylation in platinum‐resistant cells, and increased H3K9la at the promoters of RAD51 and breast cancer gene 2 (BRCA2) activates homologous recombination repair, thereby contributing to platinum resistance.^39^ In the same panel, GCN5 also catalyses RAD51 lactylation at lysine 73, which enhances RAD51 recruitment to DNA damage sites and further strengthens homologous recombination efficiency. In addition, H3K18la has been implicated in ferroptosis resistance: in tumour cells, H3K18la facilitates transformer‐2 protein homolog alpha (TRA2A)‐mediated alternative splicing of SCL/TAL1‐interrupting locus (STIL), increasing the STIL‐L isoform and thereby suppressing ferroptosis and reducing cisplatin sensitivity.^90^

Notably, the contribution of lactylation to therapy resistance is not restricted to a single drug class, but extends from platinum resistance to poly(ADP‐ribose) polymerase (PARP) inhibitor resistance. The PARP inhibitor resistance region shown in Figure 5B. In niraparib‐resistant ovarian cancer, glycolytic activation and lactate accumulation induce H4K12la, which activates super‐enhancer upstream RAD23 homolog A (RAD23A) and recruits MYC proto‐oncogene (MYC, thereby enhancing nucleotide excision repair capacity and promoting PARP inhibitor resistance.^41^ Together, these histone and non‐histone lactylation events reinforce a DNA repair‐centred resistance program in ovarian cancer. Consistent with this model, integrative transcriptomic analyses further suggest that resistant subpopulations enriched for ALDH1A1 and S100A4 expression are associated with lactylation‐related chemoresistance signatures, although this association remains inferential rather than a direct demonstration of lactylation at single‐cell resolution.^90^ Beyond tumour cell‐intrinsic repair pathways, lactate‐associated signalling also remodels the tumour microenvironment. In ovarian cancer, tumour‐derived lactate has been shown to induce H3K18la in macrophages and promote M2‐like polarisation, thereby releasing CCL18 to enhance tumour growth and metastasis.^91^ Another study showed that FOXK1 regulated glycolipid metabolism and lipid metabolism mediates TOX‐induced histone lactylation and promotes CD8^+^ T‐cell exhaustion, thereby contributing to immune modulation in HGSOC.^92^ These findings suggest that histone lactylation in ovarian cancer is not confined to malignant cells, but may extend to immune populations within the tumour microenvironment.

Upstream metabolic compensation provides an additional layer of support for this resistant phenotype. Under glucose‐limited conditions, acetyl‐CoA acetyltransferase 1 (ACAT1)‐mediated acetylation of malic enzyme 2 (ME2) at lysine 156 enhances glutamine anaplerosis and sustains pyruvate and lactate production under metabolic stress.^93^ Rather than representing a lactylation event itself, this pathway appears to support lactate‐linked resistance by maintaining the metabolic substrate supply required for downstream epigenetic remodelling. Consistently, further studies indicate that ACAT1 promotes platinum resistance by increasing ME2 acetylation, whereas sirtuin 4 (SIRT4 counteracts this process. Pharmacological inhibition of ACAT1 reduces ME2 acetylation and sensitises ovarian cancer cells to platinum‐based therapy, with synergistic activity observed in patient‐derived xenograft models. The specific pathway is illustrated in Figure 5C. Building on this metabolic–epigenetic framework, Jin et al.^94^ demonstrated that Tanshinone I suppresses glycolytic enzyme expression, reduces lactate production and down‐regulates H3K18la, thereby inhibiting ovarian cancer cell growth. Although the precise downstream oncogenic targets of H3K18la in this setting remain to be fully defined, These findings support a tighter model in which lactate‐driven lactylation acts downstream of metabolic rewiring to promote therapeutic resistance, while upstream metabolic interventions may offer a complementary strategy for restoring drug sensitivity.

## 4 TRANSLATIONAL THERAPEUTIC OPPORTUNITIES AND CURRENT LIMITATIONS

The therapeutic relevance of the lactate–lactylation axis is increasingly being explored, but the current landscape remains heterogeneous in mechanistic specificity and translational maturity. At present, most available strategies do not selectively target lactylation itself; rather, they modulate upstream lactate production, lactate transport or broader epigenetic regulators that may secondarily affect lactylation status. Therefore, the therapeutic implications of these approaches should be interpreted with caution. In the current evidence base, the most plausible clinical value may lie in biomarker‐guided combination strategies rather than in stand‐alone lactylation‐directed therapies. Representative therapeutic approaches that may modulate the lactate–lactylation axis, either directly or indirectly, are summarised in Table 1, with particular attention to translational maturity and gynaecologic relevance.

**TABLE 1:** Representative therapeutic strategies targeting lactylation‐related pathways and their current clinical or preclinical status. This table summarises candidate agents that may modulate the lactate–lactylation axis directly or indirectly. Because most current approaches affect lactate production, lactate transport or broader epigenetic/metabolic regulators rather than lactylation itself, the table distinguishes mechanistic plausibility from disease‐specific translational readiness. It highlights pharmacological limitations relevant to female reproductive diseases.

Agent	Target/mechanism	Effect on lactate/lactylation	Translational maturity	Potential gynaecologic relevance	Main limitation
Fargesin	Natural lignan; targets PKM2 and suppresses glycolytic flux towards lactate production	May reduce lactate production and downstream H3 histone lactylation by limiting PKM2‐dependent glycolytic substrate flow	Preclinical only	May be mechanistically relevant to PKM2‐overexpressing ovarian and endometrial cancer models, but direct validation in female reproductive diseases is lacking^95^, ^96^	Evidence for lactylation control derives mainly from non‐gynaecologic tumour models; natural compound pharmacokinetics, bioavailability, target selectivity, optimal formulation and potential off‐target effects on normal ovarian/endometrial metabolism require systematic evaluation.
AZD3965	Selective MCT1 inhibitor	Blocks MCT1‐dependent lactate transport and disrupts lactate flux; in MCT1‐dependent cells, this may induce intracellular lactate accumulation, acidification and secondary disturbance of lactate‐linked epigenetic signalling.	Early clinical oncology development; gynaecologic application remains hypothetical	May be relevant to ovarian cancer and other lactate‐dependent tumours, particularly in metabolically defined subgroups^97^, ^98^	Activity is likely restricted to MCT1‐high/MCT4‐low or lactate‐transport‐dependent contexts; MCT4 compensation, metabolic heterogeneity, ocular/cardiac monitoring requirements and lack of gynaecologic clinical validation remain major barriers.
Syrosingopine	Dual MCT1/4 inhibitor; repurposing candidate	Blocks lactate export through MCT1/MCT4 and may impair NAD^+^ regeneration, especially under mitochondrial stress or in combination with metformin	Preclinical/repurposing concept	May help overcome MCT4‐mediated escape from MCT1‐selective inhibition in glycolytic gynaecologic tumours^99^	Not lactylation‐specific; systemic dual MCT blockade may affect normal lactate shuttling in reproductive tissues; gynaecologic efficacy and safety remain unvalidated.
Givinostat (ITF2357)	Broad‐spectrum HDAC inhibitor; may affect HDAC1/3‐related chromatin and metabolic–enzyme acetylation pathways	May indirectly reduce lactyl donor availability by altering glycolytic‐enzyme acetylation and glycolytic flux; context‐dependent suppression of H3K18la has been proposed in treatment‐resistance settings	Approved for a non‐oncologic indication; gynaecologic relevance remains preclinical.	May be relevant to platinum‐resistant ovarian cancer and other gynaecologic malignancies through epigenetic reprogramming and resensitisation to cytotoxic therapy^100^	Not lactylation‐specific mechanistic attribution remains indirect because HDAC inhibition also affects acetylation and broader chromatin regulation; gynaecologic evidence remains preclinical.
Metformin	Biguanide; metabolic modulator linked to AMPK activation, mitochondrial homeostasis and SIRT3‐associated delactylation pathways	May restore SIRT3‐related delactylation and reduce H3K18la‐associated inflammatory or oxidative‐stress signalling; direct evidence for metformin‐driven delactylation in female reproductive diseases remains limited.	Clinically established drug with repurposing potential; lactylation‐related application remains exploratory.	May be relevant to PCOS‐associated mitochondrial/metabolic dysregulation and to selected endometrial hyperplasia or early endometrial cancer settings^101^	Lactylation‐related effects are indirect and context dependent; it remains unproven whether metformin improves PCOS or endometrial pathology through SIRT3‐mediated delactylation rather than through broader metabolic remodelling.
Demethylzeylasteral (DML)	Natural triterpenoid; reported to suppress H3K9la/H3K56la in liver cancer stem‐cell models and to promote USP22‐dependent PD‐L1 degradation	May reduce selected histone lactylation marks and suppress stemness‐ or immune‐evasion‐associated programs in preclinical models; targets USP22 and inhibits glycolysis‐related signalling	Preclinical only	Conceptually, may have relevance to stemness‐associated relapse tumour phenotypes and to immunometabolic targeting strategies in gynaecologic malignancies^102^, ^103^	Evidence remains limited to non‐gynaecologic preclinical systems; pharmacokinetics, bioavailability, formulation feasibility, tissue selectivity and potential off‐target effects in hormone‐responsive reproductive tissues remain undefined. Disease‐specific validation in female reproductive cancers is lacking.

From a cross‐disease perspective, several core regulatory nodes may be viewed as pan‐disease components of the lactate–lactylation axis, particularly LDHA, MCT family transporters and SIRT3‐related delactylation pathways. LDHA promotes lactate generation at the metabolic source, MCTs regulate intracellular and extracellular lactate distribution, and SIRT3 may contribute to the removal of selected lactylation marks or to broader restoration of metabolic homeostasis. However, the therapeutic implications of targeting these nodes are unlikely to be uniform across benign and malignant conditions. In malignant diseases such as endometrial and ovarian cancer, inhibition of lactate production or transport may be desirable to impair proliferation, disrupt tumour–stromal metabolic coupling, reverse immune suppression and sensitise tumours to chemotherapy or ferroptosis‐inducing strategies. By contrast, in benign disorders such as EM and PCOS, the objective may be less complete metabolic blockade than selective normalisation of aberrant lactate signalling, because glycolysis, lactate shuttling and mitochondrial adaptation also participate in normal endometrial remodelling, follicular development and reproductive physiology. Therefore, the same regulatory node may represent a cytotoxic target in cancer but a homeostasis‐restoring target in non‐malignant disease, with important implications for dosing, therapeutic window and biomarker‐guided patient selection.

### 4.1 Direct lactylation‐targeting strategies

From a mechanistic standpoint, direct modulation of the enzymatic machinery that installs or removes lactylation marks represents the most conceptually straightforward, but currently least mature, therapeutic strategy. Candidate writer mechanisms include p300/CBP‐associated lysine acyltransferase activity, the ACSS2/lactyl‐CoA/KAT2A axis, KAT8‐mediated pan‐lactylation reported in tumour models, and the more recently identified AARS1/2‐mediated ATP‐dependent, lactyl‐CoA‐independent lactylation pathway.^3^, ^21^, ^104^, ^105^ Conversely, lactylation may be removed by class I HDACs, particularly HDAC1–3, and by selected sirtuin‐family delactylases, including SIRT1, SIRT3 and SIRT6.^4^, ^33^, ^106^, ^107^ However, these enzymatic nodes differ substantially in the strength of mechanistic evidence, substrate specificity, disease context and pharmacologic tractability. This distinction is especially important for translational interpretation.

Among candidate writer‐directed strategies, p300/CBP is among the most frequently discussed candidate writer‐related enzymes. They may contribute to lactylation‐associated transcriptional programs through both their acyltransferase activity and canonical co‐activator functions.^3^, ^108^ Small‐molecule inhibitors such as C646 and A‐485 therefore serve as useful chemical probes to test whether p300/CBP‐dependent acyltransferase activity contributes to disease‐relevant lactylation‐associated transcriptional programs.^109^, ^110^ In reproductive biology, this concept is supported by recent GC studies showing that FSH‐driven glycolysis promotes CREB lactylation at lysine 136, facilitates CREB/CBP/p300 complex formation and activates transcriptional programs required for GC proliferation and differentiation. The observation that C646 or oxamate suppresses granulosa‐cell proliferation, differentiation and follicular development supports a functional link among FSH‐driven glycolysis, CREB lactylation and ovarian physiology. Still, it does not by itself establish a lactylation‐specific therapeutic mechanism.^35^

Nevertheless, currently available p300/CBP inhibitors lack lactylation specificity. They are expected to concurrently influence histone and non‐histone lactylation, as well as other lysine acylation processes.^111^ Consequently, unless validated through independent approaches, including site‐specific lactylation detection, gene perturbation or rescue assays and locus‐level chromatin analysis, the biological effects observed following p300/CBP inhibition cannot be attributed to decreased lactylation levels.^112^ This concern is particularly pertinent in benign reproductive system disorders such as PCOS and EM. Given that p300/CBP is extensively implicated in hormone responsiveness and developmental transcriptional regulatory programs, systemic inhibition is more likely to evoke reproductive safety concerns than to provide a selectively targeted lactylation therapy.^113^, ^114^

Likewise, the discovery of AARS1/2 as lactate‐sensing, ATP‐dependent, lactyl‐CoA‐independent lactyltransferases has expanded the conceptual framework of lactylation control. Still, it has not yet yielded selective pharmacologic tools suitable for disease‐specific intervention.^32^ This pathway is particularly intriguing because it suggests that lactate accumulation may be coupled directly to proteome‐level Kla without requiring a canonical lactyl‐CoA donor. However, whether AARS1/2‐dependent lactylation operates in parallel with p300/CBP‐mediated chromatin lactylation, dominates under acute lactate stress or preferentially modifies non‐histone substrates remains unresolved.^21^ Thus, selective direct targeting of the lactylation machinery remains an attractive long‐term strategy. Still, at present, it should be regarded as an emerging mechanistic direction rather than an established therapeutic platform.

### 4.2 Substrate‐deprivation strategies

Compared with direct writer/eraser targeting, substrate‐deprivation strategies are mechanistically less specific but currently more plausible from a translational perspective. These approaches aim to reduce the intracellular availability of lactate or disrupt lactate flux, thereby indirectly limiting the substrate context required for lactylation. Representative examples include inhibition of MCTs, blockade of lactate dehydrogenase (LDH) and upstream suppression of glycolytic enzymes such as PKM2.

#### 4.2.1 Fargesin

Fargesin, a lignan isolated from Magnolia fargesii and related Magnoliaceae plants, has been reported to target PKM2, a key glycolytic enzyme implicated in tumour metabolic reprogramming.^95^, ^96^ PKM2 has been reported in several studies to be overexpressed in ovarian and endometrial cancers and, particularly in endometrial carcinoma, to be associated with adverse clinicopathological features or poor prognosis.^115^, ^116^ In a non‐small cell lung cancer model, fargesin was shown to target PKM2, inhibit aerobic glycolysis, reduce lactate production and suppress H3 histone lactylation, thereby inhibiting tumour growth.^95^ Compared with agents that directly target epigenetic enzymes, fargesin may act upstream by limiting the metabolic substrate supply that supports lactylation.^3^, ^95^ However, its effects on lactylation‐related pathways have not yet been validated directly in gynaecologic cancers, and its relevance to ovarian or endometrial cancer therefore remains mechanistically suggestive rather than disease‐specifically established.

In the context of this review, the potential relevance of fargesin lies in the broader concept that lactylation may be therapeutically modulated not only by writers, erasers or chromatin‐associated regulators, but also through upstream metabolic intervention. Because PKM2 is linked to glycolytic reprogramming in both ovarian and endometrial cancer, PKM2‐targeting compounds such as fargesin may represent a plausible strategy to indirectly attenuate lactate‐dependent epigenetic signalling.^116^, ^117^, ^118^ Nevertheless, direct evidence linking fargesin to lactylation control in female reproductive cancers remains lacking, and future studies will be needed to determine whether the PKM2–lactate–lactylation axis is sufficiently targetable in these disease settings.

#### 4.2.2 MCT1/4 inhibitors

MCTs are major regulators of lactate trafficking across the plasma membrane and therefore represent upstream modulators of the lactate–lactylation axis. MCT1 can mediate both lactate uptake and efflux depending on cellular metabolic state and lactate gradients, whereas MCT4 is primarily responsible for supporting lactate/H^+^ efflux in highly glycolytic or hypoxic cells, helping maintain intracellular pH homeostasis under high‐lactate‐producing conditions.

AZD3965 is a first‐in‐class and clinically advanced selective MCT1 inhibitor.^119^ By binding to MCT1 with high affinity, AZD3965 effectively blocks transmembrane lactate transport. In tumour cells that rely on MCT1 for lactate export, this blockade can lead to intracellular lactate accumulation and reduced pH. Such metabolic perturbations have been proposed to inhibit glycolytic enzyme activity and disrupt bioenergetic homeostasis, thereby contributing to reduced proliferation, cytostasis or cell death.^120^, ^121^ In the phase I trial, AZD3965 showed pharmacodynamic evidence of target engagement, but dose‐limiting toxicities at higher doses included asymptomatic ocular/electroretinogram changes, cardiac troponin elevation and acidosis, indicating that ocular and cardiac monitoring are important when targeting the MCT1 systemically.^97^, ^122^ However, a major limitation of MCT1‐selective inhibition is compensation through MCT4. In hypoxic and highly glycolytic tumour compartments, MCT4‐mediated lactate/H^+^ efflux can preserve intracellular pH homeostasis and attenuate the metabolic stress induced by AZD3965.^123^ Accordingly, tumours with an MCT1‐high/MCT4‐low phenotype may be more vulnerable to AZD3965, whereas MCT4‐high tumours are less likely to respond to MCT1 blockade alone.^121^, ^124^, ^125^ This issue is particularly relevant to ovarian cancer, where hypoxic ascites‐rich microenvironments may favour MCT4‐dependent lactate export in glycolytic tumour cells. In contrast, stromal or oxidative tumour subpopulations may rely on MCT1‐dependent lactate uptake. Therefore, AZD3965 monotherapy may be insufficient in MCT4‐high gynaecologic tumours unless combined with strategies that suppress MCT4‐mediated compensation.^86^, ^124^, ^125^, ^126^

From a therapeutic‐development perspective, two forward‐looking strategies may be considered. The first is the development of dual MCT1/4 inhibitors. Syrosingopine is a representative example of this concept, as it inhibits both MCT1 and MCT4 and has been shown to induce synthetic lethality with metformin by blocking lactate export and impairing NAD^+^ regeneration under mitochondrial stress.^99^ This strategy is conceptually attractive because it may overcome the adaptive escape route created by MCT4 up‐regulation during selective MCT1 blockade. However, syrosingopine should currently be regarded as a repurposing candidate and mechanistic probe rather than a validated lactylation‐specific therapy. The second strategy is to combine AZD3965 with MCT4‐directed inhibition. Selective MCT4 inhibitors or tool compounds, such as VB124, provide a preclinical framework for testing whether simultaneous blockade of MCT1‐dependent lactate transport and MCT4‐dependent lactate efflux can produce deeper disruption of lactate homeostasis than either approach alone.^127^ Such combinations may be especially relevant in tumours with spatial metabolic heterogeneity, where MCT1 supports lactate uptake in oxidative compartments, whereas MCT4 supports lactate export in hypoxic glycolytic regions. In addition, MCT4 blockade may have immunometabolic advantages, as reduced lactate export can alleviate lactate‐driven immunosuppression, increase intratumoural pH and enhance T‐cell infiltration and activation in preclinical models.^128^, ^129^ Therefore, combining MCT1 inhibition, MCT4 inhibition, mitochondrial inhibitors or immune checkpoint blockade may represent a rational direction for future therapeutic development.

Importantly, the translational meaning of MCT inhibition differs substantially between malignant and benign reproductive diseases. In ovarian and endometrial cancers, MCT blockade is primarily conceptualised as a cytostatic or cytotoxic strategy aimed at disrupting tumour lactate export, metabolic symbiosis, immune evasion and treatment resistance.^130^ By contrast, in EM and PCOS, the therapeutic goal would be selective normalisation of aberrant lactate signalling rather than broad suppression of lactate transport. This distinction is essential because lactate shuttling participates in normal reproductive physiology, including GC–oocyte metabolic cooperation, endometrial remodelling and decidual metabolic adaptation.^60^, ^131^, ^132^ Thus, although MCT1/4 may be relevant to the lactate‐rich inflammatory microenvironment of endometriotic lesions and to altered ovarian or endometrial metabolism in PCOS, systemic MCT inhibition in these benign disorders remains speculative and would require careful evaluation of reproductive safety, tissue selectivity, dosing window and effects on follicular development and endometrial receptivity.

#### 4.2.3 LDH inhibitors

LDH catalyses the reversible interconversion of pyruvate and lactate while coupling this reaction to NADH/NAD^+^ redox balance. By regulating lactate production, LDH directly influences intracellular lactate availability and may therefore modulate the substrate context for lactylation‐associated signalling. Isoform specificity is biologically important because LDHB‐enriched LDH‐1, which is abundant in oxidative tissues such as the heart, preferentially supports lactate‐to‐pyruvate conversion, whereas LDHA‐enriched LDH‐5 favours pyruvate‐to‐lactate conversion in highly glycolytic tissues.^133^ Thus, LDH inhibition may affect not only lactate‐producing pathological cells but also lactate‐utilising physiological tissues.

Although LDH is an intuitive therapeutic target, the development of LDH inhibitors has remained challenging. Early compounds such as gossypol and FX11 provided proof of principle for LDH‐directed metabolic intervention, but their translational utility has been limited by issues including insufficient selectivity, pleiotropy and suboptimal drug‐like properties; for gossypol, clinically recognised toxicity concerns, such as hypokalaemia, also constrained broader adoption.^134^, ^135^, ^136^ Newer agents, including GNE‐140 and its active enantiomer (R)‐GNE‐140, have improved potency, but they are better regarded as LDHA/LDHB inhibitors rather than strictly LDHA‐selective compounds. Galloflavin has also shown LDH‐inhibitory and anti‐tumour activity in endometrial cancer models, whereas (R)‐GNE‐140 has demonstrated metabolic synergy in ovarian cancer models when combined with additional metabolic stressors.^137^, ^138^

In the context of the present review, the relevance of LDH inhibition lies not only in suppressing glycolytic flux but also in its potential to reduce the lactate supply that supports lactylation‐associated signalling. This upstream strategy is conceptually attractive because it may attenuate both metabolic and epigenetic consequences of lactate accumulation. In ovarian and endometrial cancers, LDH inhibition may be exploited to reduce lactate production, disrupt redox homeostasis, impair metabolic plasticity and potentially weaken lactylation‐associated oncogenic programs. In contrast, in EM and PCOS, the goal should not be broad suppression of LDH activity; the therapeutic goal should be selective normalisation of aberrant lactate–lactylation signalling. Therefore, LDH inhibitors should currently be viewed as upstream modulators of the lactate–lactylation axis rather than lactylation‐specific therapies. Future studies should determine whether LDH inhibition reduces disease‐relevant lactylation marks such as H3K18la, H3K9la or non‐histone lactylation in a site‐specific and cell‐type‐specific manner. For malignant diseases, this should be tested in biomarker‐defined subgroups and rational combinations with chemotherapy, ferroptosis‐inducing agents, mitochondrial inhibitors or immune‐metabolic therapies. For benign disorders such as EM and PCOS, future work should prioritise reproductive safety, tissue selectivity, dosing window and preservation of physiological lactate‐dependent functions.

### 4.3 Pleiotropic drugs with secondary effects on lactylation

#### 4.3.1 Givinostat

Givinostat is a broad‐spectrum class I/II HDAC inhibitor with established clinical use outside oncology, particularly in Duchenne muscular dystrophy (DMD).^139^, ^140^ Recent reviews have discussed its possible relevance to lactylation biology, including potential effects on H3K18 lactylation.^100^ However, direct evidence demonstrating that givinostat specifically suppresses H3K18la and restores platinum sensitivity in ovarian cancer remains limited.^141^ Thus, givinostat should currently be regarded as a candidate epigenetic modulator with possible indirect effects on lactylation‐related pathways, rather than as a validated lactylation‐targeted therapy in gynaecologic cancer.^100^, ^140^ Givinostat has demonstrated efficacy in delaying the decline in muscle function in Phase III clinical trials for DMD and has secured Food and Drug Administartionm approval.^142^, ^143^ In oncology, its anti‐inflammatory and pro‐apoptotic properties make it a potential candidate for combination therapy in gynaecological malignancies. In the context of hyperthermic intraperitoneal chemotherapy or systemic chemotherapy for ovarian cancer, co‐administration of Givinostat may merit evaluation as a strategy to suppress resistant tumour cell populations. However, its side effect of inducing autophagy may counteract its cytotoxicity, suggesting that clinical applications may warrant the concurrent use of autophagy inhibitors to maximise therapeutic efficacy.

#### 4.3.2 Metformin

Metformin, a first‐line antidiabetic agent, has attracted interest for its potential anti‐tumour and immunometabolic effects. Recent mechanistic studies suggest that it may influence lactylation‐related pathways, at least in part by regulating SIRT3, a mitochondrial sirtuin with delactylase activity. In non‐gynaecologic systems, SIRT3 has been shown to delactylate the non‐histone substrate CCNE2 and suppress tumour growth; however, whether metformin exerts comparable SIRT3‐dependent delactylation effects on histone and non‐histone targets in female reproductive diseases remains to be established.^144^ In a zebrafish inflammation model, metformin reduced H3K18la, decreased reactive oxygen species production and attenuated neutrophil recruitment, supporting the concept that metformin can modulate inflammation‐associated lactylation in vivo.^145^ This mechanism may be relevant to the chronic inflammatory response within ovarian cancer ascites.

In PCOS, reduced SIRT3 expression has been reported in ovarian GCs and oocytes, together with metabolic and mitochondrial dysfunction.^146^ These alterations may contribute to impaired oocyte quality and subfertility. PCOS itself is also associated with an increased risk of endometrial cancer, although a direct causal role of SIRT3 dysregulation in this risk remains unproven. In a PCOS mouse model, metformin was shown to prevent Sirt3 down‐regulation in oocytes, and other studies suggest that metformin can improve oocyte developmental competence^101^; however, whether metformin improves fertility in PCOS specifically by preserving oocyte mitochondrial function through SIRT3 requires further direct evidence.^147^, ^148^ Given that the endometrium in patients with PCOS may exhibit altered glycolytic activity and, potentially, aberrant lactylation status,^82^ metformin may have the capacity to remodel the epigenetic landscape of preneoplastic endometrial lesions^149^; however, whether this involves restoration of SIRT3‐mediated delactylation remains to be determined.^33^ Clinical studies suggest that metformin, mainly as an adjunct to progestin‐based or combined regimens, may benefit selected patients with atypical endometrial hyperplasia or grade 1 stage IA endometrioid endometrial carcinoma. However, current evidence remains mixed.^150^, ^151^ Organic cation transporters and multidrug and toxin extrusion proteins expressed in the endometrium may contribute to local metformin handling, but this mechanism is still largely hypothetical.^152^ In this setting, repurposing clinically established agents such as metformin may be more translationally feasible in the near term than directly targeting lactylation.

#### 4.3.3 Demethylzeylasteral

Demethylzeylasteral (DML), a triterpenoid isolated from *Tripterygium wilfordii*, has attracted attention as a natural compound that may influence lactate‐related epigenetic regulation. The strongest evidence so far comes from non‐gynaecologic preclinical models. In liver cancer stem cells, DML reduced selected histone lactylation marks, including H3K9la and H3K56la, accompanied by lower expression of stemness‐associated genes, impaired sphere formation and reduced tumourigenicity.^102^ These findings suggest that DML may interfere with lactate‐supported stemness programs, but they do not establish DML as a lactylation‐specific inhibitor.^153^ Indeed, its biological effects are likely to reflect broader metabolic and epigenetic changes. Separately, DML has been reported to target the deubiquitinase USP22, promote USP22 degradation and enhance ubiquitin–proteasome‐mediated PD‐L1 degradation, indicating a potential role in tumour immune regulation through a mechanism not specific to lactylation.^103^

For female reproductive diseases, the relevance of DML remains largely inferential. In ovarian and endometrial cancers, where lactylation has been linked to stemness, immune remodelling and therapy resistance, DML is a mechanistically interesting yet unvalidated candidate for future study.^70^, ^91^ At present, there is no direct evidence that DML suppresses lactylation‐dependent progression, chemoresistance or immune escape in these malignancies. Its possible relevance to benign disorders, including EM and PCOS, is even more preliminary. Although lactate metabolism and lactylation have been implicated in inflammatory remodelling, follicular dysfunction and impaired endometrial receptivity, DML has not yet been tested in these disease‐specific contexts.^57^, ^80^ Thus, DML is best viewed as an exploratory natural compound that illustrates how lactate–lactylation‐associated pathways might be modulated indirectly, rather than as an established therapeutic candidate for benign or malignant female reproductive diseases.

### 4.4 Nanotechnology‐based therapeutic strategies

Nanotechnology‐targeted drug therapy is a treatment modality that employs nanoscale carriers to accurately deliver drugs to specific cells, tissues or organs. Currently, nanotechnology‐based approaches have been explored to improve the delivery and efficacy of agents targeting tumour metabolism, including those that may indirectly influence lactate accumulation. These strategies, such as nanoparticle‐mediated co‐delivery of chemotherapeutic agents with metabolic inhibitors or lactate‐scavenging systems, offer potential advantages in terms of pharmacokinetics and tumour targeting. To date, researchers have developed a paclitaxel (PTX)–lipid–dendrimer hybrid nanohybrid system, specifically a lipid–dendrimer hybrid nanocarrier loaded with PTX. This system showed synergistic activity in ovarian cancer models, with increased PTX sensitivity and prolonged survival.^154^ Looking ahead, a promising strategy is to co‐encapsulate LDH inhibitors and chemotherapeutic agents such as PTX. By blocking the cellular energy supply, the metabolic inhibitor would hypersensitise cancer cells to the chemotherapy.

Another strategy involves using nanomaterials to directly ‘scavenge’ lactate, rather than merely inhibiting its production. In 2020, Chen et al.^155^ ingeniously combined the unique respiratory characteristics of the facultative anaerobe *Shewanella oneidensis* MR‐1 with tumour metabolic features. Under hypoxic conditions, Shewanella can utilise lactate as an electron donor, transferring electrons to metal minerals (such as tetravalent manganese) for respiration. The research team constructed a bio‐hybrid system termed ‘Bac@MnO_2_’ by decorating the bacterial surface with manganese dioxide (MnO_2_) nanoflowers. In this system, the bacteria oxidise lactate (electron donor) for respiration, while MnO_2_ serves as the electron acceptor. This process simultaneously decomposes lactate and generates oxygen. By depleting intratumoural lactate – thereby cutting off the substrate source for lactylation – and generating oxygen to alleviate hypoxia, this approach significantly sensitises tumours to radio‐ and chemotherapy. This strategy of physical or biochemical substrate clearance may offer a potential approach for treating refractory solid tumours. However, it is important to emphasise that these approaches are not specific to lactylation per se, but rather operate at the level of lactate metabolism or the tumour microenvironment more broadly. As such, they should be viewed as supportive or enabling strategies within a wider metabolic intervention framework, rather than as direct lactylation‐targeted therapies. Their relevance to lactylation biology is therefore indirect and remains to be further defined in disease‐specific contexts.

Taken together, these findings indicate that the lactate–lactylation axis may have translational relevance across benign and malignant female reproductive diseases. However, the maturity of the available evidence varies substantially across disease entities. To distinguish mechanistic interest from translational readiness, the key candidate markers, targetable pathways, biospecimens and major barriers to clinical application are summarised in Table 2.

**TABLE 2:** Translational relevance of the lactate–lactylation axis in female reproductive diseases. This table summarises the key candidate biomarkers, targetable pathways, clinically relevant biospecimens and major barriers. Particular emphasis is placed on assay standardisation, disease‐ and compartment‐specific validation, pan‐disease biospecimen feasibility and experimental strategies required to distinguish causal lactylation events from downstream markers of metabolic rewiring.

Disease	Candidate biomarker	Targetable pathway	Sample	Main barrier/next step	Key references
Endometriosis	H3K18la; LDHA/PDK1; METTL3–HIF‐1α–HMOX1 axis	Glycolysis; lactate‐associated H3K18la; METTL3–HIF‐1α–HMOX1‐mediated ferroptosis resistance; macrophage remodelling	Lesion tissue; matched eutopic endometrium; peritoneal fluid; peritoneal macrophages	Causality remains uncertain; disease specificity is unclear; lesion subtype and peritoneal microenvironment are heterogeneous; standardised assays for site‐specific H3K18la detection are lacking; future studies should combine lesion‐based IHC/CUT&Tag with paired peritoneal‐fluid analyses and locus‐specific perturbation models.	^52^, ^53^, ^54^, ^55^, ^56^, ^57^, ^58^, ^59^, ^60^, ^61^
Endometrial cancer	H3K18la; USP39; lactate‐associated TAM markers	H3K18la–USP39–PI3K/AKT/HIF‐1α signalling; TAM polarisation; context‐dependent ferroptosis/stress‐response programs	FFPE tumour tissue; fresh tumour tissue; tumour immune microenvironment; plasma as exploratory comparator	Requires large, well‐annotated FFPE cohorts with standardised IHC or spatial profiling of H3K18la/USP39; prognostic or predictive value should be tested independently of FIGO stage, histological subtype, molecular classification, treatment response and other clinicopathological variables; causality should be evaluated using locus‐specific H3K18la perturbation models.	^67^, ^68^, ^69^, ^70^, ^71^, ^72^
PCOS	CREB K136la; H3K9la/H3K18la; PKM2‐associated lactylation signatures	FSH/CREB‐related lactylation; PKM2‐associated glycolysis; steroidogenic and folliculogenic reprogramming; endometrial receptivity‐associated lactylation	Granulosa cells; follicular fluid; endometrial tissue; peripheral blood or serum metabolic markers as exploratory non‐invasive comparators	Requires phenotype‐stratified validation across PCOS subtypes, BMI/insulin‐resistance status, androgen excess, ovulatory status and treatment history; paired follicular‐fluid, granulosa‐cell, endometrial and circulating‐marker analyses are needed to determine whether local lactylation signals can be approximated by less invasive samples; causal effects should be tested using genetic or locus‐specific epigenetic perturbation.	^35^, ^73^, ^74^, ^75^, ^76^, ^77^, ^78^, ^79^, ^80^, ^81^, ^82^
Ovarian cancer	H3K9la/H3K18la; H4K12la; RAD51K73la; MCT1/MCT4	DNA damage repair; ferroptosis resistance; lactate transport; glycolytic plasticity; tumour–immune metabolic crosstalk	Tumour tissue; ascites; ascites‐derived tumour cells and immune cells; paired plasma as exploratory comparator	Requires compartment‐resolved validation in tumour cells, ascites macrophages and T‐cell populations; biomarker studies should distinguish lactylation‐driven resistance from broader glycolytic adaptation, DNA repair activation and MCT1/MCT4‐dependent lactate transport; predictive cutoffs should be tested in treatment‐response cohorts.	^39^, ^40^, ^41^, ^84^, ^85^, ^86^, ^87^, ^88^, ^89^, ^90^, ^91^, ^92^, ^93^, ^94^, ^95^
Cross‐disease axis	H3K18la; MCT1/MCT4; LDHA; SIRT3; lactate‐related metabolic signatures	Glycolysis; lactate transport; site‐specific histone/non‐histone lactylation; delactylation/homeostatic reprogramming machinery	Disease‐relevant tissue/fluid; ascites, peritoneal fluid, follicular fluid, endometrial/tumour tissue; peripheral blood or plasma as low‐invasive exploratory biospecimens requiring paired validation	Shared upstream nodes are potentially targetable across diseases, but their downstream consequences differ substantially between benign and malignant contexts. Key next steps include validating site‐specific assays for H3K18la/H3K9la with ChIP‐seq‐ or CUT&Tag‐grade antibodies; defining whether lactylation can be reliably measured in peripheral blood or plasma, or whether tissue‐, ascites‐, peritoneal‐fluid‐, follicular‐fluid‐ or lesion‐based profiling is required; conducting paired biospecimen studies to assess concordance between circulating and local lactylation signals; establishing disease‐specific biomarker cutoffs and therapeutic windows; and using locus‐specific perturbation models, such as dCas9‐based recruitment of erasers, to test causality.	^3^, ^4^, ^22^, ^33^, ^34^, ^35^, ^38^, ^87^, ^88^, ^89^, ^97^, ^105^, ^106^

Abbreviation: FFPE, formalin‐fixed paraffin‐embedded.

## 5 DISCUSSION

The studies reviewed here place the lactate–lactylation axis at the interface between metabolic rewiring and epigenetic regulation in female reproductive diseases.^3^ Across EM, endometrial cancer, PCOS and ovarian cancer, abnormal lactate accumulation appears to be associated with histone and non‐histone lactylation events that may influence transcriptional regulation, immune remodelling, ferroptosis sensitivity, steroidogenic dysfunction and therapeutic response.^39^, ^54^, ^70^, ^80^ These observations argue against viewing lactylation simply as a metabolic byproduct; instead, lactylation may help transmit metabolic stress into disease‐relevant transcriptional and cellular programs.^3^

The strength of this evidence, however, varies sharply across diseases and experimental systems. Most mechanistic insights come from cell‐based systems or animal models, whereas validation in human tissues and independent clinical cohorts remains limited. In addition, the maturity of the evidence differs substantially across disease entities. Ovarian and endometrial cancers currently provide the strongest mechanistic evidence, particularly for links between lactylation, DNA damage repair, ferroptosis resistance, macrophage remodelling and oncogenic transcription.^39^, ^67^, ^70^, ^80^ By contrast, the roles of lactylation in EM and PCOS are supported by fewer disease‐specific studies, and its contribution to lesion persistence, inflammatory remodelling, steroidogenic dysfunction or impaired folliculogenesis remains less clearly defined.^54^, ^67^, ^80^, ^156^ Accordingly, conclusions regarding disease causality, biomarker utility and therapeutic relevance should be interpreted with appropriate caution.

A recurring theme across these studies is that lactylation is highly context dependent. Lactylation should not be regarded as a uniform biological consequence of lactate accumulation. The disease setting, the cellular compartment, local oxygen and lactate gradients, transporter expression and the modified substrate shape its consequences. These factors may partly explain why lactylation is linked to pro‐tumourigenic transcriptional programs in some settings yet shows distinct or apparently divergent effects in others.^3^, ^12^, ^67^, ^70^ Importantly, elevated lactate should not be equated with pathogenic lactylation perse, because lactate accumulation may also reflect broader changes in glycolytic flux, acidosis, redox balance and microenvironmental remodelling.^12^, ^157^

Several methodological barriers still limit interpretation. The first is assay standardisation. Although immunoblotting, immunofluorescence, immunohistochemistry, ChIP‐qPCR, ChIP‐seq and mass spectrometry have all been used to detect lactylation, these platforms are not yet standardised for clinical use or cross‐study comparisons.^3^, ^38^ A particular concern is antibody specificity for site‐defined histone lactylation marks. Antibodies recognising H3K18la, H3K9la, H3K56la or other Kla sites may differ in affinity, cross‐reactivity and suitability across applications.^158^, ^159^ An antibody that performs adequately in immunoblotting may not be reliable for immunohistochemistry or ChIP‐based assays. Therefore, future studies should report antibody validation in detail, including peptide competition assays, dot blots against related acyl‐lysine modifications, orthogonal confirmation by mass spectrometry when feasible and application‐specific validation for tissue staining or chromatin profiling.^160^, ^161^ The development of validated, ChIP‐seq‐grade antibodies for key marks such as H3K18la and H3K9la will be essential before lactylation can be robustly linked to locus‐specific transcriptional regulation in patient tissues.^38^, ^158^

Human validation should also become more disease‐specific and clinically structured. For malignant diseases such as endometrial and ovarian cancer, future studies should use large, independent and well‐annotated patient cohorts to perform immunohistochemical analysis of candidate marks such as H3K18la, H3K9la or H4K12la.^162^, ^163^, ^164^ These analyses should determine whether lactylation markers are independently associated with survival, recurrence, treatment response or chemoresistance, regardless of standard clinicopathological variables, including stage, grade, histological subtype, molecular classification, residual disease and treatment regimen.^163^, ^164^ In parallel, paired analysis of tumour tissue, adjacent non‐tumour tissue, ascites, stromal compartments and immune cell populations would help determine whether lactylation is a tumour cell‐intrinsic event, a microenvironmental response or both. For benign diseases such as EM and PCOS, the cohort design should account for menstrual phase, hormonal treatment, lesion subtype, ovarian stimulation status, insulin resistance, body mass index and inflammatory or metabolic comorbidities, all of which may influence lactate metabolism and lactylation readouts.^39^, ^91^, ^165^

A further priority is to move beyond correlation. Many current studies show that lactate accumulation and lactylation marks change together, but this does not establish whether a specific lactylation event is instructive. Future preclinical studies should combine metabolic perturbation with site‐ and locus‐selective approaches.^166^, ^167^, ^168^ For example, dCas9‐based recruitment of delactylases or engineered eraser domains to defined promoters or enhancers could be used to reduce H3K18la or H3K9la at selected loci without globally suppressing glycolysis.^33^, ^107^ Conversely, rescue experiments using lactylation‐deficient or lactylation‐mimetic mutants of non‐histone targets, such as CREB or RAD51, would help distinguish direct lactylation‐dependent effects from broader metabolic or chromatin changes.^35^, ^39^, ^107^ Such approaches would be particularly valuable for testing whether H3K18la at the METTL3, USP39 or TP53 loci is causally required for ferroptosis resistance, oncogenic transcription or stress‐induced tumour suppression.

Biomarker development also needs to consider sample accessibility. For EM, lesion tissue remains the most informative specimen for mapping site‐specific lactylation, but it requires surgery and is unsuitable for repeated monitoring. Peritoneal fluid may better reflect the inflammatory, lactate‐rich microenvironment of the lesion, whereas peripheral blood would be more clinically feasible but may be less disease‐specific.^169^, ^170^, ^171^ For PCOS, GCs and follicular fluid obtained during assisted reproduction provide direct access to the ovarian microenvironment, but they are not practical for routine diagnosis or longitudinal monitoring.^172^, ^173^, ^174^ Peripheral blood, serum lactate‐related metabolites, circulating immune‐cell lactylation or extracellular vesicle‐associated markers may be more feasible to measure. Still, their relationship to ovarian or endometrial lactylation must be validated carefully.^175^, ^176^ Thus, future biomarker studies should compare tissue, local fluid and blood‐based readouts in the same patients to determine which specimens best capture disease‐relevant lactylation biology.^177^

Therapeutically, the field remains at an early stage. Preclinical studies suggest that modulation of lactate production, lactate transport or lactylation‐related enzymes may have therapeutic relevance, particularly when combined with chemotherapy, immunotherapy or metabolic intervention. Yet most proposed interventions act upstream of lactylation or affect broad epigenetic programs rather than selectively targeting lactyl marks. This distinction is critical because HDAC inhibitors, metformin, MCT inhibitors, LDH inhibitors and lactate‐scavenging approaches may alter lactylation‐associated readouts without demonstrating that their biological effects are lactylation dependent. This distinction is particularly important when interpreting the therapeutic significance of agents such as HDAC inhibitors, metformin, MCT inhibitors, LDH inhibitors or lactate‐scavenging nanomaterials.^97^, ^119^, ^178^, ^179^

Repurposing approved metabolic drugs may be more realistic in the near term than developing lactylation‐specific agents. Metformin is the most relevant example because it is already widely used for metabolic indications and has been clinically explored in PCOS and in endometrial hyperplasia or early endometrial cancer, often in combination with progestin‐based fertility‐sparing regimens.^180^, ^181^, ^182^, ^183^ However, these studies were not designed to test lactylation as a mechanism or biomarker. In future trials, it would be useful to incorporate exploratory endpoints such as H3K18la immunohistochemistry in endometrial tissue, lactate‐related metabolites in serum or local fluids and SIRT3‐related delactylation markers where biologically justified. Such studies could clarify whether the clinical effects of metformin involve restoration of metabolic homeostasis alone, modulation of lactylation‐associated signalling or both.

This problem is especially evident in gynaecological malignancies, where lactate metabolism may differ between primary tumours, metastatic deposits, ascites‐associated tumour cells, stromal compartments and immune populations.^184^, ^185^, ^186^ The biological consequences of lactate accumulation may differ not only among patients but also between primary tumours, metastatic lesions, stromal compartments and immune cell subsets within the same patient. This heterogeneity may partly account for variable responses to approaches targeting lactate transport or glycolytic metabolism. Future translational studies should therefore define the metabolic and lactylation status of specific tumour compartments rather than extrapolating directly from bulk tumour profiles, non‐gynaecological models or single disease settings.^187^

In EM and PCOS, the problem is different. Here, the key question is not how to suppress proliferating tumour cells, but whether abnormal lactate‐linked signalling disrupts tissue homeostasis, inflammation, steroidogenesis or follicular development.^54^, ^80^, ^81^ This distinction is important because glycolysis, lactate shuttling, mitochondrial adaptation and chromatin remodelling are all involved in normal endometrial cycling, decidualisation, follicular growth, granulosa‐cell differentiation, luteinisation and oocyte competence.^188^, ^189^, ^190^, ^191^, ^192^ Broad inhibition of glycolysis, MCT‐mediated lactate transport, HDAC/sirtuin activity or p300/CBP‐dependent acylation could therefore disrupt physiological ovarian and endometrial programs.^33^, ^189^, ^193^ Future studies in benign disease models should evaluate reproductive safety endpoints, including ovulation, luteal function, endometrial receptivity, implantation, embryo development and fertility, rather than relying only on lesion size, inflammatory markers or cellular proliferation.^147^, ^188^, ^191^

In these settings, lactylation may represent a mechanistic link among metabolic dysregulation, inflammatory remodelling and reproductive dysfunction. At the same time, the available evidence is still insufficient to determine whether lactylation acts primarily as a disease driver, a context‐dependent modifier or a downstream marker of altered metabolism.^54^, ^80^, ^81^, ^156^ Clarifying this issue will be essential before lactylation‐directed interventions can be considered in benign gynaecological diseases.

At a broader level, the lactate–lactylation axis shows both shared and disease‐specific features across female reproductive diseases. Across EM, endometrial cancer, PCOS and ovarian cancer, enhanced glycolytic flux and lactate accumulation represent recurring upstream conditions, and regulatory nodes such as H3K18la, LDHA, MCTs and SIRT3‐related delactylation pathways may define a shared mechanistic framework with potential translational relevance.^3^, ^33^, ^39^, ^54^, ^70^ The same upstream metabolic pressure may therefore be read out differently across diseases: as ferroptosis resistance and immune remodelling in EM, oncogenic or stress‐adaptive transcription in endometrial cancer, steroidogenic and follicular dysfunction in PCOS and DNA repair‐centred drug resistance in ovarian cancer.^39^, ^41^, ^54^, ^70^, ^80^ These observations suggest that common upstream regulators may provide candidate pan‐disease targets, but their therapeutic relevance is unlikely to be uniform. In malignant diseases, inhibition of lactate production or transport may help suppress tumour‐promoting signalling, disrupt metabolic crosstalk and improve treatment sensitivity.^39^, ^41^, ^141^ By contrast, in benign disorders such as EM and PCOS, a more realistic goal may be to normalise aberrant metabolic–epigenetic signalling rather than broadly suppress lactate metabolism.^188^, ^190^, ^192^

In summary, the lactate–lactylation axis offers a plausible framework for linking metabolic reprogramming to epigenetic regulation in female reproductive diseases.^3^ Current evidence supports its mechanistic relevance across EM, endometrial cancer, PCOS and ovarian cancer, but the maturity of evidence remains heterogeneous and is still weighted towards preclinical models.^39^, ^54^, ^70^, ^80^ The next step is not simply to identify more lactylation‐associated changes, but to determine which of them are causal, measurable and clinically informative. This will require standardised, site‐specific lactylation assays; validation in human samples; clearer definition of reader‐mediated mechanisms; careful assessment of tissue‐ and fluid‐based biomarkers; and rational testing of approved metabolic drugs in studies that include lactylation‐relevant endpoints. These efforts will determine whether the lactate–lactylation axis can progress from an emerging mechanistic concept to a clinically useful platform for biomarker development and therapeutic intervention.

## AUTHOR CONTRIBUTIONS

Jiajun Qiao: conceptualisation, literature investigation, writing and original draft. Yue Xiao: literature investigation, writing, review and editing. Yi Cheng: literature investigation, writing, review and editing. Weihai Xu: writing, review and editing. Jing Shu: supervision, writing, review and editing. Shishi Li: conceptualisation, supervision, writing, review and editing. All authors read and approved the final manuscript.

## CONFLICT OF INTEREST STATEMENT

The authors declare no conflicts of interest.

## FUNDING INFORMATION

This work was supported by grants from Zhejiang Province Traditional Chinese Medicine Science and Technology Project (GZY‐ZI‐KJ‐23058).

## ETHICS STATEMENT

The authors have nothing to report.

## CONSENT

The authors have nothing to report.

## Data Availability

No new datasets were generated or analysed in this study.
